# Size‐Related Electrochemical Performance in Active Carbon Nanostructures: A MOFs‐Derived Carbons Case Study

**DOI:** 10.1002/advs.201901517

**Published:** 2019-08-21

**Authors:** Srinivas Gadipelli, Zhuangnan Li, Yue Lu, Juntao Li, Jian Guo, Neal T. Skipper, Paul R. Shearing, Dan J. L. Brett

**Affiliations:** ^1^ College of Physics Sichuan University Chengdu 610064 China; ^2^ Electrochemical Innovation Lab Department of Chemical Engineering University College London Torrington Place London WC1E 7JE UK; ^3^ Department of Chemistry University College London 20 Gordon Street London WC1H 0AJ UK; ^4^ Department of Physics & Astronomy University College London London WC1E 6BT UK; ^5^ The Faraday Institution Quad One Harwell Science and Innovation Campus Didcot OX11 0RA UK

**Keywords:** carbon nanostructures, metal–organic frameworks (MOFs)/zeolitic imidazolate frameworks (ZIF‐8), oxygen reduction reaction (ORR), particle size–related performance, supercapacitors

## Abstract

Metal–organic framework–derived carbon nanostructures have generated significant interest in electrochemical capacitors and oxygen/hydrogen catalysis reactions. However, they appear to show considerably varied structural properties, and thus exhibit complex electrochemical–activity relationships. Herein, a series of carbon polyhedrons of different sizes, between 50 nm and µm, are synthesized from zeolitic imidazolate frameworks, ZIF‐8 (ZIF‐derived carbon polyhedrons, ZDCPs) and their activity is studied for capacitance and the oxygen reduction reaction (ORR). Interestingly, a well‐correlated performance relationship with respect to the particle size of ZDCPs is evidenced. Here, the identical structural features, such as specific surface area (SSA), microporosity, and its distribution, nitrogen doping, and graphitization are all strictly maintained in the ZDCPs, thus allowing identification of the effect of particle size on electrochemical performance. Supercapacitors show a capacity enhancement of 50 F g^−1^ when the ZDCPs size is reduced from micrometers to ≤200 nm. The carbonization further shows a considerable effect on rate capacitance—ZDCPs of increased particle size lead to drastically reduced charge transportability and thus inhibit their performance for both the charge storage and the ORR. Guidelines for the capacitance variation with respect to the particle size and SSA in such carbon nanostructures from literature are presented.

## Graphical abstract

**Carbon‐based nanostructures** are indispensable for supercapacitors, fuel cells, and batteries. Advancement in this area requires critical understanding of the form and function. Here, a well‐correlated relationship is demonstrated for the performance of supercapacitors and the oxygen reduction reaction with respect to particle size of metal–rganic frameworks–derived carbon. Surface area, pore‐size distribution, nitrogen doping, and graphitization are all maintained identical.

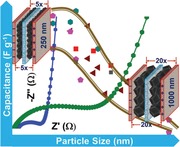

## 1 Introduction

Carbon‐based porous nanostructures are indispensable for electrochemical energy storage and conversion devices, e.g., in supercapacitors, fuel cells, and batteries.^1^, ^2^, ^3^, ^4^, ^5^, ^6^, ^7^, ^8^, ^9^, ^10^ Efforts have been made to understand the structure‐related performance characteristics of these active carbons. The desirable structural features of such carbons include: 1) accessible surface/or capacity to accommodate guest species—which is a combination of specific surface area (SSA) and porosity, 2) surface heterogeneity, and 3) electrical conductivity. In electrochemical energy storage and conversion applications, a high amount of charge adsorption/accumulation and distribution/separation is desirable. For instance, in supercapacitors, the extensive accumulation and physical charge separation of electrolyte ions is a desired criterion. This particular property is significantly influenced by the porous structure of active carbon used in the electrodes. Thus, this area is quite rigorously explored and there exists an established relationship. Here, the electrical double‐layer (EDL) capacitance tends to increase proportionally with respect to the SSA (hereafter denoted as SSA_BET_) of the carbons when comparing isostructures.^11^, ^12^ The surface heterogeneity and conductivity of the carbons also play a major role in determining overall performance.^1^, ^2^, ^3^, ^4^, ^5^, ^6^, ^7^, ^8^, ^9^, ^10^, ^11^, ^12^, ^13^, ^14^, ^15^ This is by facilitating the adsorption and distribution of ionic charges (in the case of supercapacitors), and reactants and products (in the case of catalysis reactions). It is established that nitrogen, and certain other heteroatom and transition metal–related dopants, in the carbons create charged (heterogenous) surface regions.^15^, ^16^, ^17^, ^18^ Thus, these dopants act as strong binding sites and facilitate enhancement of the electrochemical activity.

In most of the cases, the carbon‐based nanostructures are fabricated via high‐temperature thermolysis (also referred to as pyrolysis, carbonization, or graphitization) and/or chemical vapor deposition of molecular building blocks. These include biomass, metal–organic frameworks (MOFs), polymers, and templates.^1^, ^2^, ^3^, ^4^, ^5^, ^6^, ^7^, ^8^, ^9^, ^10^, ^11^, ^12^, ^13^, ^14^, ^15^, ^16^, ^17^, ^18^, ^19^ In particular, the MOFs, by nature crystalline and porous solids built upon organic linker molecules with coordinated metal nodes, have shown great potential in readily delivering multifunctional nanocarbons.^1^, ^2^, ^3^, ^4^, ^5^, ^6^, ^9^, ^10^, ^14^, ^15^, ^16^, ^17^, ^18^, ^19^, ^20^, ^21^ Such MOFs exhibit highly accessible SSA_BET_ for guest molecular species, with a well‐connected and open‐framework topology. Another advantage of such MOFs is that they are highly reproducible, and there is a standardized protocol for lab‐ or industrial‐scale synthesis, and are commercially available. More specifically, ZIFs (zeolitic imidazolate frameworks), such as ZIF‐8 analogs/prototypes are easily synthesized under mild conditions or at room temperature. Synthesis can be performed using common laboratory washing solvents such as methanol, by stirring, microwave, continuous flow, vapor, or solid‐state routes.^14^, ^15^, ^16^, ^17^, ^18^, ^21^, ^22^, ^23^, ^24^ The ZIF‐8, formed by 2‐methyl imidazolate (CH_3_C_3_H_2_N_2_) linkers with the ligand heteroatoms (C and N) and single‐metal‐atom (Zn) in the form of Zn–N_4_ coordination, exhibits a highly microporous framework with a significant surface area up to 2000 m^2^ g^−1^. These properties, along with the volatile nature of zinc metal at above 900 °C, makes ZIF‐8 one of the most explored precursor materials in the development of functional carbon nanoporous structures.^1^, ^2^, ^3^, ^4^, ^5^, ^6^, ^9^, ^15^, ^16^, ^17^, ^18^, ^21^, ^22^, ^23^, ^24^, ^25^ Such materials are often referred to as MOFs‐/ZIFs‐derivatives or in general MOFs‐derived carbons (MDCs/ZDCs).

These MOFs‐ and ZIFs‐derived nanostructures have opened up broad interest and new directions in the development of active structures for numerous applications. These include porous solids for molecular sorption, storage, and separation, heterogenous catalysis, and electrochemical energy conversion and storage.^1^, ^2^, ^3^, ^4^, ^5^, ^6^, ^9^, ^10^, ^14^, ^15^, ^16^, ^17^, ^18^, ^19^, ^20^, ^21^, ^22^, ^23^, ^24^, ^25^, ^26^, ^27^, ^28^ Every year, an exponentially growing number of such products are explored. Thermolysis of MOFs can deliver highly porous carbon nanostructures, with simultaneous incorporation of intrinsic ligand heteroatoms and metal centers within the carbon matrix, and metal oxide nanostructures or combinations thereof. Such structures are intensively investigated for oxygen and hydrogen catalysis reactions (ORR, OER, and HER—oxygen reduction, evolution, and hydrogen evolution reactions; the main reactions in fuel cells, metal–air batteries, and water electrolyzers), and supercapacitors. The advantage of this approach is that functional and conducting carbons can be easily developed through the rational design of MOFs. Accordingly, ZIF‐8 deserves a special mention. With the choice of enriched volatile (e.g., Zn) or stable (e.g., Fe, Co, Ni, Pt) metal centers, or addition of such metal–inorganic complexes, the ZIFs can generate metal‐free or metal‐incorporated nitrogen‐doped carbons, with a significant degree of graphitization.^1^, ^2^, ^3^, ^4^, ^5^, ^6^, ^14^, ^15^, ^16^, ^17^, ^18^, ^21^, ^22^, ^23^, ^24^, ^25^, ^29^, ^30^ Numerous publications report the use of ZIF‐based derivatives for electrochemical capacitors and electrocatalysis, which is an order magnitude higher than any other MOF‐derivatives explored for such purposes. However, it is worth noting that the derivatives, either carbon, metal‐oxides, or combinations of both, produced from the same precursor, ZIF‐8, under similar thermolysis conditions, show considerable variation in their electrochemical activity performance.^18^, ^31^, ^32^, ^33^, ^34^, ^35^, ^36^, ^37^, ^38^, ^39^, ^40^, ^41^, ^42^, ^43^, ^44^, ^45^, ^46^, ^47^, ^48^, ^49^, ^50^, ^51^, ^52^ This ambiguous nature is also observed in other types of MOFs, including polymeric and biomass precursors.^1^, ^2^, ^3^, ^4^, ^5^, ^6^, ^7^, ^8^, ^9^, ^10^, ^11^, ^12^, ^13^, ^53^, ^54^, ^55^, ^56^, ^57^, ^58^, ^59^ Therefore, there is an urgent need to understand the relationship between form and function to advance this class of materials and develop bespoke productions routes.

**Figure 1:**
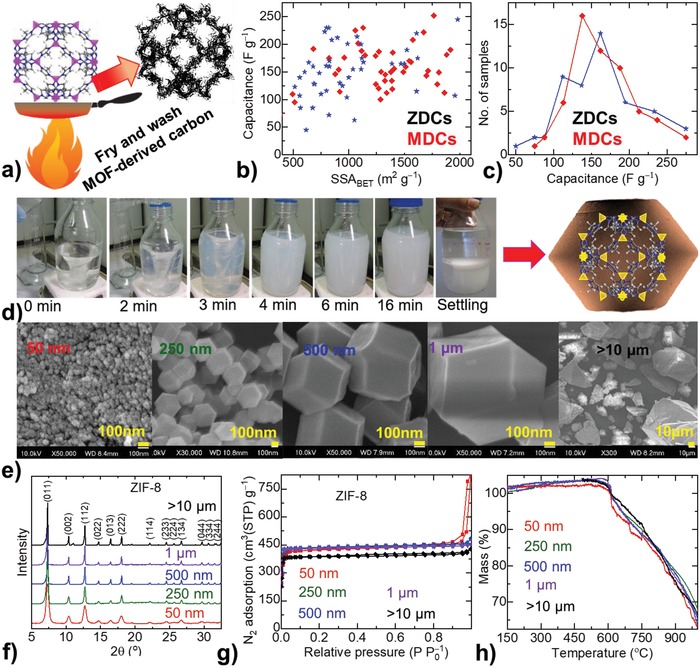
a) Production of MOFs‐derived carbons via pyrolysis. b) Overview of the literature reported capacitance values of over 100 carbon samples related to MDCs (red colored data) and ZDCs (blue colored data) against their SSA_BET_. c) Capacitance distribution with respect to the number of samples. See Table S1 in the Supporting Information for the full details on the synthesis conditions of MDCs/ZDCs, particle size, porosity, capacitance with associated test conditions, and related reference works. d–h) Synthesis and structural characteristics of ZIF‐8 samples with varied size of polyhedrons in this work: d) Methanol solution‐based synthesis at room temperature and schematic representation of imidazolate linker‐zinc metal center framework assembly and pore structure—the tetrahedrons in yellow are for the Zn–N_4_ coordination, and carbon, nitrogen, and hydrogen atoms of the imidazolate linker are represented by gray, blue, and white color spheres. e) SEM micrographs, from left to right show increased particle size on batch‐wise. f) PXRD patterns indicate the highly crystalline nature. g) N_2_ adsorption–desorption isotherms inform top quality framework porosity structure. h) Thermogravimetric curves confirm that all the samples are in their highest quality.

As represented in **Figure** **1**a–c, and Table S1 in the Supporting Information, these MDCs/ZDCs evidence significant difference in their EDL capacitance by up to 100 F g^−1^ at a given SSA_BET_. This equates to more than 50% “uncertainty” for the given, more realistic, capacitance value of ≈150 F g^−1^ (Figure 1b,c). This is clearly seen from their capacity distribution with respect to the number of samples studied. The samples with SSA_BET_ in the range of 500–2000 m^2^ g^−1^ are selected as more acceptable. Note that the samples show more or less constant EDL capacitance values even though the SSA_BET_ is varied over quite a large extent. Such a trend is far more complex than the generally accepted proportional relation between EDL capacitance and porosity (including both microporosity and SSA_BET_ of the carbons). In such cases, this behavior is attributed to the porous structure itself. The pore widths and their distribution in the microporous (pore widths of ≤2 nm) to mesoporous (pore widths between 2 and 50 nm) region have shown quite distinctly different charge storage capacities. Similarly, the porosity associated with functionalized surfaces, for instance, N‐doping, has also been found to define the capacitance in the structures. However, as shown in Figure 1b, and Table S1 in the Supporting Information, the ZDCs designed under similar synthesis conditions are expected to have the same porosity and surface features, but show a large difference in their charge storage capacity.^18^, ^31^, ^32^, ^33^, ^34^, ^35^, ^36^, ^37^, ^38^, ^39^, ^40^, ^41^ For example, the ZDCs produced at the same carbonization temperature of 900 °C, show no correlation between their specific capacitance value and SSA_BET_.^18^, ^31^, ^32^, ^33^, ^34^, ^35^ The same is also observed for the ZDCs derived at 950 or 1000 °C.^35^, ^36^, ^37^, ^38^, ^39^, ^40^, ^41^ A possible reason for this is differences in the particle size of the carbon nanostructures. As summarized in Table S1 in the Supporting Information, in most cases the samples show quite different particle sizes for the reported capacitance values. The particle size of the carbons varied by a large extent between a few tens of nanometers to µm scale. Due to the varied laboratory environments and synthesis conditions/concentrations of constituents, the ZIFs/MOFs or other bottom‐up molecular precursor structures precipitate with a varied size of particles/architectures. This is mostly maintained in their carbonized products.^1^, ^2^, ^3^, ^4^, ^5^, ^6^, ^7^, ^8^, ^9^, ^10^, ^11^, ^12^, ^13^, ^14^, ^15^, ^16^, ^17^, ^18^, ^19^, ^20^, ^21^, ^22^, ^23^, ^24^, ^25^, ^26^, ^27^, ^28^, ^29^, ^30^, ^31^, ^32^, ^33^, ^34^, ^35^, ^36^, ^37^, ^38^, ^39^, ^40^, ^41^, ^42^, ^43^, ^44^, ^45^, ^46^, ^47^, ^48^, ^49^, ^50^, ^51^, ^52^, ^53^, ^54^, ^55^, ^56^, ^57^, ^58^, ^59^, ^60^, ^61^, ^62^ This important size–property relationship is generally overlooked compared to the routinely highlighted and explored porosity and/or N‐doping effects in the MDCs/ZDCs.

Therefore, herein, ZIF‐8 is selected as a prototype precursor to elucidate the derived porous carbon nanostructures particle size effect on the electrochemical performance variations, particularly for EDL capacitance and ORR. A series of up to five batches of ZIF‐8 samples are prepared. It is well known that ZIFs (and most MOFs) exhibit a well‐defined composition, structure, and crystallinity, with polyhedron crystals of uniform size. Here, the synthesis conditions are controlled to produce varied crystallite sizes of ZIF‐8 polyhedrons from 50 nm to µm. Later, the ZIF‐8 samples are directly carbonized, without further surface modification and/or addition of extra heteroatom precursors, at the most favorable temperatures of 900 and 1000 °C.^15^, ^20^, ^21^, ^24^, ^25^, ^26^, ^30^ The ZIF‐derived carbon polyhedrons (ZDCPs) are then examined for all the structural and electrochemical properties, without further acid washing or chemical treatment, e.g., KOH activation (Table S1, Supporting Information). Interestingly, the ZDCPs produced at a particular temperature, but from the different batches, exhibit isostructural characteristics, such as a similar degree of graphitization, and amount of nitrogen doping and porosity. However, a significant performance difference is observed in their electrochemical capacitance and oxygen catalysis. For example, the symmetric supercapacitors in aqueous electrolyte (6 m KOH) exhibit the highest capacitance for the nanosized ZDCPs, with particle size < 200 nm. The increase in particle size of ZDCPs tends to show gradually decreased capacitance. Up to 50 F g^−1^, less capacity is observed for the ZDCPs of µm size. Furthermore, the carbonization temperature shows a profound effect on the rate performance, where the increased particle size yields rapidly deteriorated capacitance. A similar particle size dependence of ORR performance is also observed. A detailed discussion is provided for the size‐dependent electrochemical properties through their charge/electron transfer characteristics. The data collection from literature is presented for supercapacitor charge storage against porosity and particle size, and established the guidelines for further understanding of the relevant capacity variations.

## 2 Results and Discussion

The synthesis and characterization of ZIF‐8 crystals are presented in Figure 1d–h. ZIF‐8 samples of five batches are synthesized to exhibit a varied size range of polyhedron crystals, and are thoroughly characterized by scanning and transmission electron microscopy (SEM and TEM), powder X‐ray diffraction (PXRD), Fourier transformed infrared spectroscopy (FTIR), surface area and porosity isotherms, and thermogravimetry (see The Experimental Section). Samples are named according to their polyhedron crystal sizes, observed by SEM and TEM, as ZIF‐8: 50 nm, 250 nm, 500 nm, 1 µm, and >10 µm (Figure 1e and Figure S1, Supporting Information). Due to their nanosized crystals, the 50 nm sample exhibits broader PXRD peaks (Figure 1f).^24^ A step‐like nitrogen adsorption isotherm of this nanosized sample is directly attributed to the external mesopores formed by nanoparticles (Figure 1g).^14^, ^24^, ^28^ However, all the samples exhibit the same framework structure, porosity and thermal stability, as observed in the PXRD, FTIR, surface area/porosity analysis, and thermogravimetry (Figure 1d–h, and Figure S2 and Table S2, Supporting Information). Note that the samples with crystal sizes between 50 nm and 1 µm are synthesized at room temperature by stirring the methanolic solutions of precursors in a controlled fashion (Figure 1d). The ZIF‐8 sample of greater than 10 µm sized crystals is obtained via a solvothermal synthesis route using DMF (*N*,*N*‐dimethylformamide) solvent.^30^ This route is known to yield less pure ZIF‐8, as is seen from comparative PXRD and FTIR data. Thus, the sample shows somewhat less porosity and varied particle sizes. The ZIF‐8 samples produced from the methanolic solvent route at room temperature show the highest quality structure with uniform crystal sizes.^14^, ^15^, ^24^

**Figure 2:**
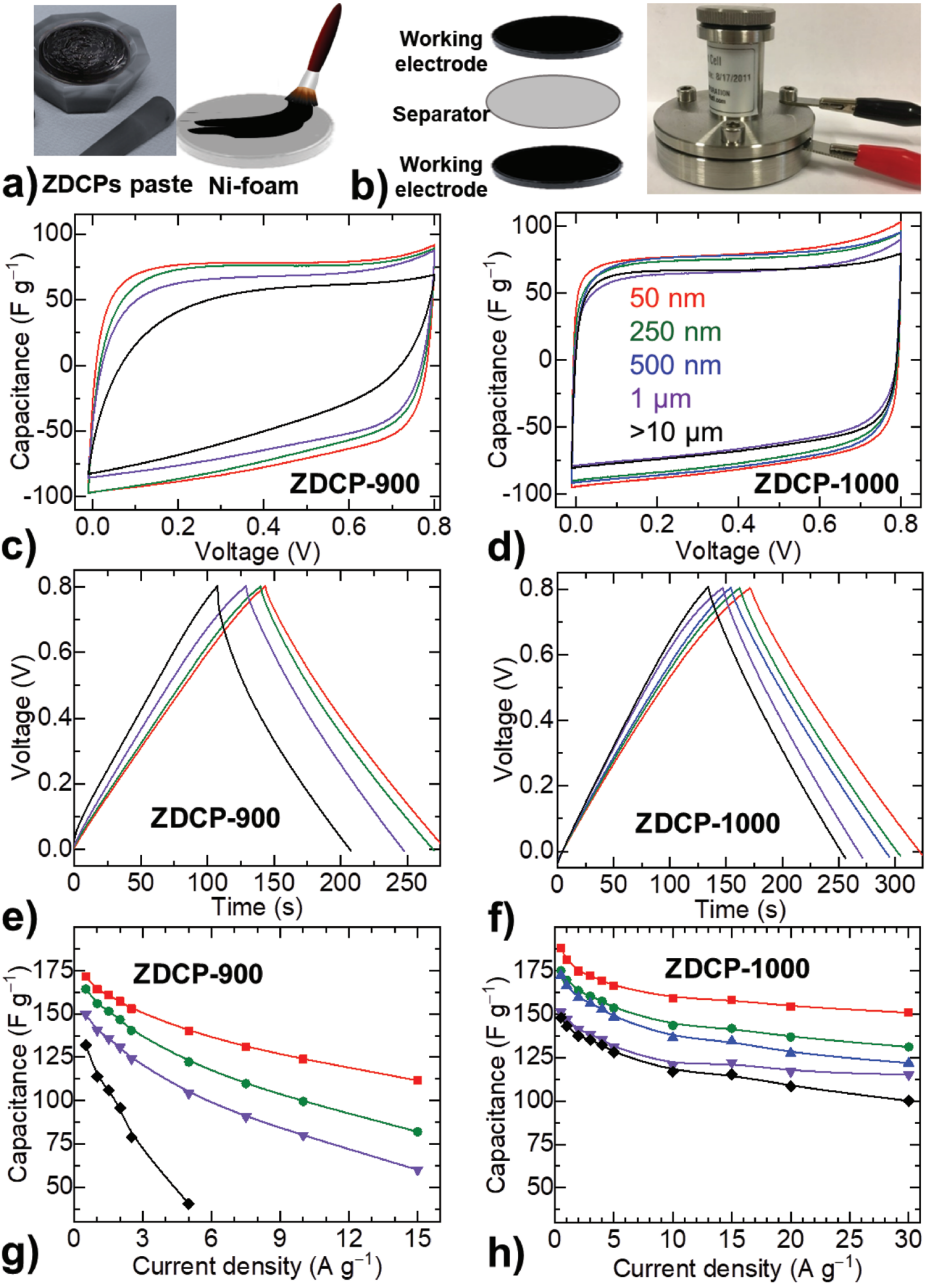
ZDPCs‐based supercapacitors and their energy storage characteristics: a,b) Schematic representation of electrode preparation and two‐electrode symmetric supercapacitor device assembly used in this study. c,d) CV curves, measured at a scan rate of 20 mV s^−1^. e,f) GCD curves, measured at 0.5 A g^−1^. g,h) Rate capacitance curves, the capacitance deduced from the discharge curves at different applied current densities. Same color code applies for the data in all panels. See the Supplementary Information for additional experimental data on CV and GCD, and also rate capacitance curves deduced from the CV at different scan rates. The left and right panels represent the data from ZDCP‐900 and ZDCP‐1000 samples, respectively.

Next, the ZDCPs are produced by direct pyrolysis of ZIF‐8 samples at 900 and 1000 °C (ZDCP‐900 and ZDCP‐1000)—found to be the ideal temperature region and most commonly explored synthesis condition. Note that the ZDCPs derived at lower carbonization temperatures exhibit poor electrochemical activities.^25^, ^35^, ^60^, ^61^, ^62^ As‐derived ZDCPs are then directly examined, without further acid washing or surface modification, for the electrochemical energy storage by using two‐electrode symmetrical supercapacitor test cells (**Figure** **2** and Figures S3–S6, Supporting Information). The electrodes are made by roller‐casting a paste of active material (ZDCPs with 10 wt% PTFE, polytetrafluoroethylene binder) onto the nickel foam disks without the addition of conducting carbon, e.g., Ketjen or acetylene black. The capacitance performance is tested in alkaline electrolyte (6 m KOH) using cyclic voltammetry (CV), galvanostatic charge–discharge (GCD) cycles, and impedance tests. The characteristic shapes of CV and GCD curves, in a near rectangular and isosceles triangle, respectively, indicate EDL capacitance behavior. In both cases, the area under curves is a representative measure of the energy storage capacity. The same principle applies to understand the capacitance values from GCD curves. According to the capacitance calculation, *C* = (*I* × *t*)/Δ*V* (in F g^−1^; where *I* is the applied current density in A g^−1^, *t* is the time taken for full charge or discharge in seconds, and *V* is the operating voltage window in V), in a fixed voltage range the capacitance values are directly determined by the time. Normally, the discharge curve is considered to be the more reliable measure of capacitance. The electrode polarization during the charging process normally yields much longer charge time. As shown in Figure 2c–f, the ZDCPs of increased particle size lead to a net decrease in the capacitance value. Both the CV and GCD curves at a particular scan rate and current density, respectively, show a similar trend of particle size to capacitance (Figures S3–S6, Supporting Information).

**Figure 3:**
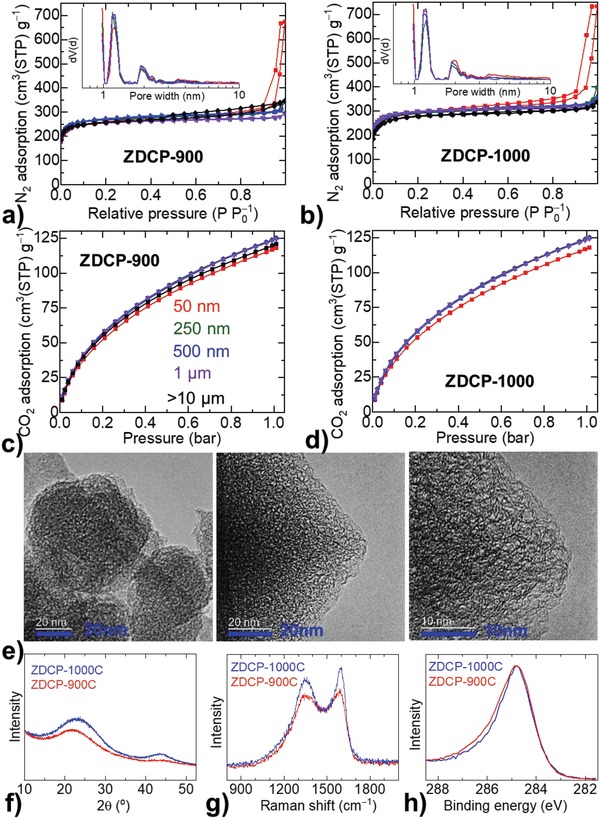
Structural characteristics of ZDCPs: a,b) N_2_ adsorption–desorption isotherms, with inset as pore‐size distribution curves, measured at 77 K. c,d) CO_2_ uptake isotherms, measured at 273 K. Same color code applies for the data in panels (a)–(d). e) TEM micrographs of ZDCP‐1000 for a size of 50 nm (at left) and 250 nm (middle and right). f–h) PXRD patterns, and Raman and XPS C 1s spectra of ZDCPs (50 nm) carbonized at 900 °C (red data) and 1000 °C (blue data).

**Figure 4:**
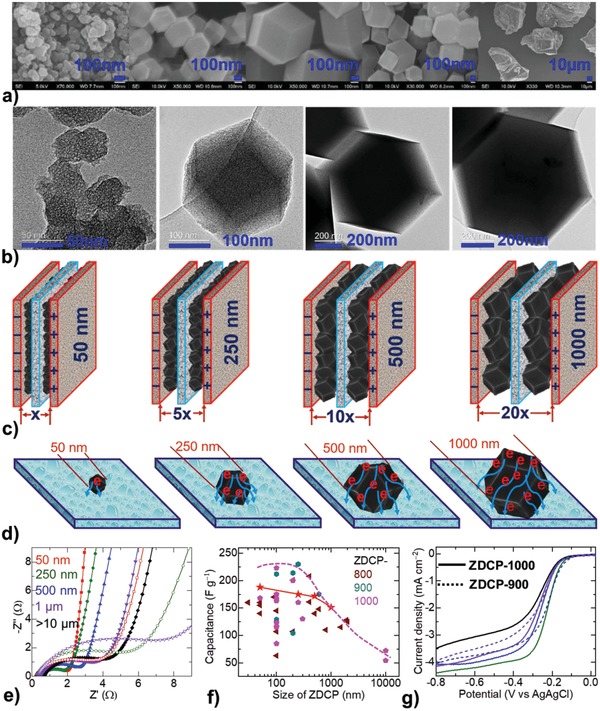
Elucidation of size effects—associated mechanism and properties: a,b) SEM and TEM micrographs of ZDCP‐1000 samples at different sizes. c) Supercapacitor packs assembly at increased carbon particle size. d) Influence of carbon particle size on the charge collector electrode for supercapacitor and electrocatalysis applications, with the associated resistive path and increased electronic path length to the charge collector. e) Impedance Nyquist curves of ZDCP‐1000 (solid data) and ZDCP‐900 (open symbol) series samples. f) Size dependence variation of capacitance in the ZDCPs—the data reproduced from the literature are grouped together with the same color for a particular synthesis temperature, and the red star data are in this study. The dotted line is a guide boundary to show the capacitance limitation/variation with respect to the increased particle size. Refer to Table S1 in the Supporting Information for the full details. g) LSV curves for ORR—same color code applies here as in (e). See Figures S9 and S10 in the Supporting Information for further details on the supercapacitor impedance and ORR catalytic parameters.

Furthermore, a significant influence of the particle size in their rate capacitance behavior can be seen (Figure 2g,h and Figures S3–S6, Supporting Information). Here the larger particles tend to show rapidly decreased rate capacitance. This property is further considerably affected by the graphitization degree/temperature. It is worth noting that the increased carbonization temperature leads to the enhanced graphitization (i.e., electrically more conductive—as discussed fully in the following and **Figures** **3** and **4**). Accordingly, the ZDCP‐900 with increased particle size exhibits rapid loss in their rate capacitance. A highly improved rate capacitance performance is seen in the ZDCP‐1000.

In order to elucidate these performance differences, the ZDCPs are critically examined to assess their structural and chemical characteristics. For this, the as‐produced ZDCPs are thoroughly characterized by various complementary techniques, such as porosity, CO_2_ adsorption, electron microscopy, PXRD, Raman spectroscopy, and X‐ray photoemission spectroscopy (XPS; Figure 3, and Figures S7 and S8 and Tables S3 and S4, Supporting Information). It is interesting to see that the N_2_ and CO_2_ uptake isotherms show similar adsorption volume indicating the similar porosity nature in ZDCPs of various particle sizes, derived at a particular temperature (Figure 3a–d). The deduced porosity parameters, such as SSA_BET_, cumulative pore volume, and micropore volume are summarized in Table S3 in the Supporting Information. The samples exhibit SSA_BET_ of about 1000 and 1100 m^2^ g^−1^ for ZDCP‐900 and ZDCP‐1000, respectively.^31^, ^35^ The qualitative behavior of the N_2_ isotherms indicates the characteristic Type‐1 isotherms, and suggesting the predominant microporous samples. The pore‐size distribution curves inform that all the pores are situated in the microporous region (≤2 nm pore widths) and all samples show a very similar trend (insets of Figure 3a,b, and Figure S7, Supporting Information). Three different pore widths are associated with the precursor ZIF‐8 framework porosity; a major portion of pores at ≈1.2 nm, second prominent pores at < 1 nm, and third and small fraction of pores at ≈2.0 nm.^15^, ^24^, ^28^ The largest fraction of pores in the 1.2 nm region is the same size as the cavity in the starting precursor ZIF‐8, indicating that the actual ZIF‐8 open framework is preserved in the ZDCPs.^28^ Those additional narrow slit‐like pores of less than 1 nm and the new pore formation in the 2 nm region are attributed to the framework collapse within the ZIF‐8 cavities and decomposition/evaporation of nitrogen/related carbon and zinc species.^10^, ^15^, ^21^, ^24^, ^25^, ^26^, ^30^

CO_2_ adsorption isotherms in the low‐pressure region also imply the highly microporous nature of the carbon samples (Figure 3c,d).^10^, ^15^, ^19^, ^24^, ^25^, ^26^, ^27^, ^28^, ^30^, ^53^ The identical CO_2_ uptake behavior in the ZDCP‐900 or ZDCP‐1000 samples further indicating the similar level of porosity and concentration of heteroatoms, e.g., N‐doping. The N‐doping can increase the basicity of the carbon structure thus leads to a stronger binding of CO_2_ molecules within the pore surface via Lewis‐acid/Lewis‐base interactions.^19^ This effect can be further understood from the CO_2_ uptakes normalized to the surface areas.^24^, ^25^ Accordingly, the increased CO_2_ capacity in the ZDCP‐900 of somewhat less SSA_BET_ informs the higher level of N‐content in the samples relative to ZDCP‐1000 series.^15^, ^19^, ^24^, ^25^, ^26^, ^27^, ^28^, ^30^ The exact amount and nature of N‐doping in the samples are discussed in the following XPS results section. The microporous and weak graphitic nature of carbon can be seen from TEM micrographs (Figure 3e). This is further supported by the PXRD, Raman, and XPS data (Figure 3f–h, and Figure S8, Supporting Information). It is worth noting that the ZDCP (‐900 or ‐1000) samples of different particle sizes show very similar graphitization features. The increased graphitization is observed for ZDCP‐1000 series samples, compared to the ZDCP‐900 series, with corresponding enhancement in their characteristic peak signal intensity. For instance, the emerging diffraction peak intensities at 25° and 44° for (002) and (100) or (101) planes, respectively, indicate the onset formation of graphitic carbon–carbon planes and in‐plane crystallinity from a poorly ordered graphitic structure in ZDCP‐900. This is also supported by Raman spectra with a prominent sp^2^ C associated G‐band (at ≈1600 cm^−1^), and the intense defects induced D‐band (at ≈1350 cm^−1^) (Figure 3 and Figure S8, Supporting Information). Considerably broadened D and G bands and featureless second‐order bands (2D and G+D) between 2700 and 3000 cm^−1^ indicate a disordered carbon network, as evidenced by PXRD and TEM.

XPS elemental analysis gives further information about the graphitization and nitrogen doping in the samples. The comparative individual C 1s, N 1s, and Zn 2p elemental core‐level spectra of the samples are presented in Figure 3h (Figure S8 and Table S4, Supporting Information). The temperature‐induced decomposition and evaporation of ligand H, C, and N species and coordinated‐zinc metal centers lead to varied chemical interactions between the remaining C, N, and Zn atoms.^14^, ^15^, ^24^, ^25^, ^26^, ^27^, ^28^, ^29^, ^30^ This is evidenced by corresponding peak shifts and narrowing or broadening in the C 1s, N 1s, and Zn 2p spectra. The exact nature of the interactions is identified through the deconvolution and fitting of C 1s and N 1s peaks (Figure S8, Supporting Information). C 1s profile of ZDCP‐900 is mostly attributed to the graphitic sp^2^ C (peak positioned at ≈284.6 eV) with the shoulder peak at ≈287 eV for the N‒C and/or Zn‒N‒C coordination.^14^, ^15^, ^24^, ^25^, ^26^, ^27^, ^28^, ^29^, ^30^ An enhanced graphitization with the loss of N‐/Zn‐content is seen in ZDCP‐1000. The N 1s peaks situated at ≈398.6 and ≈400.6 eV are assigned to pyridinic and pyrrolic type nitrogen, respectively. Weak pyrrolic nitrogen in the ZDCP‐900 samples is attributed to the remaining Zn–N coordination.^24^ The initial ZIF‐8 shows a narrow symmetric peak at ≈399.4 eV for one type of nitrogen in the framework. The elemental quantification indicates a high level of nitrogen doping, about 10 atom%, in the ZDCP‐900 samples, and is reduced to about 3 atom% in the ZDCP‐1000 samples.^18^, ^34^ About 1 atom% and trace amount of zinc is detected in ZDCP‐900 and ZDCP‐1000 samples, respectively. It is worth noting that a similar composition is formed for all the samples synthesized at a particular carbonization temperature (Table S4, Supporting Information). The structural transformation with decomposition steps associated with the evaporation of N‐ and C‐species (on the ligand linker) and Zn‐metal centers during the carbonization of ZIFs/MOFs is well documented in the literature.^15^, ^20^, ^21^, ^24^, ^25^, ^26^, ^27^, ^28^, ^29^, ^30^ It reveals a major decomposition followed by reconstruction and N‐doping occurs when the carbonization temperature is increased to greater than 800 °C. The thermogravimetric and XPS data presented in Figure 1h and 3h, and Figure S8 in the Supporting Information directly support this.

All the above structural information clearly suggests that the ZDCPs produced at a particular temperature show identical structural parameters, such as the amount of porosity, nitrogen doping, and graphitization (Figure 3, and Figures S7 and S8 and Tables S3 and S4, Supporting Information). This is the first report to maintain such isostructural characteristics in the ZDCPs of varied sizes. Previous works show significant variation in the porosity, e.g., in this context, with respect to their particle size/dimension (Table S1, Supporting Information).^10^, ^31^, ^32^, ^33^, ^34^, ^35^, ^36^, ^37^, ^38^, ^39^, ^40^, ^41^, ^42^, ^43^, ^44^, ^45^, ^46^, ^47^, ^48^, ^49^, ^50^, ^51^, ^52^ It is worth noting that the ZDCPs of different particle size in a single literature report also show varied porosity.^42^, ^43^, ^44^ For example, ZDCPs with a size of 90, 600, and 1900 nm yield an SSA_BET_ of 825, 736, and 693 m^2^ g^−1^ and the symmetric capacitors deliver the specific capacitance of 170, 148, and 128 F g^−1^ at 1 A g^−1^ in 6 m KOH electrolyte.^42^ Although the increased capacity is observed with respect to the SSA_BET_ of the ZDCPs, here, the authors attribute the superior charge storage capacity in the smaller ZDCPs to the facile penetration of electrolyte ions into the deep pores. In our work, one should expect a constant capacitance from such isostructural ZDCPs within the ZDCP‐900 or ZDCP‐1000 series. However, as presented in Figure 2, and Figures S3 and S4 in the Supporting Information, the samples show a significant and systematic variation in their capacitance values with respect to the increased particle size of the carbon polyhedrons. Another important thing to note is that ZDCPs of the same size exhibit improved performance for both the capacity and rate capacitance, in agreement with the increased porosity (Figures 2 and 3, and Table S3, Supporting Information). It is proposed that N‐sites can also contribute to the psuedocapacitance.^13^, ^21^, ^57^ As the series of ZDCPs produced at a particular temperature exhibits identical N‐content, this is not influencing on their comparative capacitance trend.

Figure 4 depicts a further mechanism to explain the effect of particle size on electrochemical activity, with the representative active carbon polyhedrons assembled on the electrode collector plates. As shown in Figure 4c,d, the increased carbon particle size experiences the long‐range charge transport paths to reach the charge collector plate/electrode. Thus, the efficient charge storage and transport mechanism is highly dependent on the electrical conductivity of the carbon particle. Accordingly, the ZDCPs derived at low graphitization temperature are expected to show poor activity, as increase in the particle size contributes to a more resistive path for charges to be stored/distributed/transported. The ionic and electronic charge separation, distribution, and transportation are strongly determined by the electrical conductivity and porosity of the active carbon structures. This is evidenced in their characteristic ion transport impedance curves (Figure 4e, and Figure S9 and Table S5, Supporting Information). The samples are increasingly resistive with respect to the increased particle size. This can be observed in the cases of both ZDCP‐900 and ZDCP‐1000 series of samples. Samples produced at high carbonization temperature, ZDPC‐1000, show highly improved ion transport characteristics. The particle size dependence variation in their charge transport characteristics is also largely minimized compared to the ZDCP‐900 series. This is evidenced by the shorter semicircle and larger slopes, and are associated with a smaller charge transfer resistance and better capacitive behavior, respectively. The ZDCP‐900 samples show significantly increased resistance for charge transport (*R*_ct_) with respect to the increased particle size of carbons (Table S5, Supporting Information). For example, in the case of 50 nm particles, this *R*_ct_ is increased from 1.35 Ω in ZDCP‐1000 to 4.6 Ω in ZDCP‐900. Bode plots also indicate rapid frequency response for the smaller particles size carbons. The relaxation time constants, τ_0_ (= 1/ƒ_0_), of 1.1 and 1.4 s for 50 and 500 nm in the ZDCP‐1000 samples are significantly smaller than those of 4.6 and 8.2 s from ZDC‐900 with respective particle sizes of 50 nm and 1 µm. The critical frequency ƒ_0_ at a phase angle of −45° represents the point where the capacitive and resistive impedances are equal.

Furthermore, Figure 4f,g demonstrates this particle size defined the activity variation for the EDL capacitance and ORR. Critical analysis of the literature data shows a clear relationship between carbon particle size and capacitance in the ZDCPs. The ZDCPs produced at three different temperatures in the literature are grouped together to show the exact influence of the particle size on the capacitance. In all cases, the samples show a net decrease in their capacitance when the particle size of the ZDCPs is greater than 200 nm (Figure 4f and Table S1, Supporting Information). Such a trend is also observed in their ORR activity. The activities are reported by standard linear sweep voltammetry (LSV) tests conducted in O_2_‐saturated 0.1 m KOH using a rotating disk electrode (RDE) configuration (Figure 4g and Figure S10, Supporting Information). The ideal ORR catalyst, e.g., Pt/C, exhibits a step‐like LSV curve.^14^, ^15^, ^16^, ^17^, ^18^ The more positive reduction potential, steep slope (also known as minimum half‐wave potential), and a large current response (limiting current density) are the main required characteristics for efficient ORR activity. Accordingly, the LSV curves of the ZDCP‐900 and ZDCP‐1000 series samples tend to show a considerable activity variation among the samples: unfavorable ORR activity characteristics with respect to the increased carbon particle size. In addition, the level of N‐doping as well as graphitization plays a critical role in enhancing the electron transport characteristics.^14^, ^15^, ^25^, ^30^ ZDCP‐1000 samples show smaller Tafel slopes and larger electron transfer number, *n*, with faster reaction kinetics and efficient reaction pathway (Figure S10, Supporting Information). For instance, ZDCP‐1000 of 250 nm shows Tafel slope of ≈103 mV dec^−1^ and *n* of ≈3.3, whereas it is increased to ≈131 and ≈144 mV dec^−1^ for ZDC‐1000 of 500 nm and ZDCP‐900 of 1 µm, respectively. Pt/C exhibits the dominant four‐electron pathway (i.e., O_2_ is reduced to OH^−^). This four‐electron reaction pathway is favorable compared to the two‐electron for efficient ORR activity. Accordingly, the samples with large particle sizes show sluggish ORR activity. The steeper slopes in the kinetic region, with a correspondingly smaller Tafel slope for the nanosized carbons, indicate the enhanced ORR activity. Overall, for samples of the same size, the enhanced porosity can boost the ORR activity as it can facilitate O_2_ adsorption, binding sites, and distribution of reactants and products.^3^, ^15^, ^25^ The high level of nitrogen doping in the ZDCP‐900 samples shows favorable onset reduction potentials compared to their ZDCP‐1000 counterparts. The active basic N‐sites in the porous carbons can create positive cores for the oxygen binding to enhance the ORR catalytic activity. In addition, the relatively high concentration of N‐components, graphitic‐N and pyridinic‐N, and the degree of graphitization and high porosity should contribute to the enhancement of the overall electrocatalytic performance.^14^, ^15^, ^25^

## 3 Conclusion

The effect of carbon nanostructure particle size on the electrochemical charge storage capacitance and ORR is demonstrated. For this, a series of isostructural ZDCPs with varied sizes, 50 nm, 250 nm, 500 nm, 1 µm and > 10 µm, are designed and their isostructural features maintained, such as similar SSA_BET_, identical porosity and pore‐size distribution, same concentration of N‐doping, and equal degree of graphitization. This is the first report in which a systematic study, maintaining such characteristics has been applied to ZDCPs, allowing identification of the effect of particle size on electrochemical performance. A gradual decrease in the charge storage capacity and ORR activity with respect to the increased size of ZDCPs is observed. The symmetric supercapacitors evaluated under conditions of best practice show about 50 F g^−1^ capacity enhancement initially in the ZDCPs with a size less than 200 nm compared to the µm‐sized particles. A further significantly widened capacity difference is seen in their rate performance at increased current densities. Among the samples, the smaller particles of ZDCPs show highly reduced charge transfer resistance and better capacity behavior. This property is affected by the degree of ZDCP graphitization—where the ZDCPs derived at 900 °C show drastically reduced performance with the increased particle size. The critical analysis of the literature data shows a well‐defined trend and net capacity decrease, more than 50 F g^−1^, with the increased particle size to a µm range. Furthermore, the capacity variation against the SSA_BET_ and capacity distribution among the MOFs‐ and ZIFs‐derived carbon nanoporous samples is depicted. This shows the specific capacitance of about 150 F g^−1^ is the most accepted/populated among the samples.

## 4 Experimental Section

*Synthesis of ZIF‐8 Samples—ZIF‐8 (Sample 50 nm and 250 nm)*: The two different sizes of ZIF‐8 samples were obtained by adding solution in different order.^15^, ^24^ The zinc nitrate hexahydrate Zn(NO_3_)_2_⋅6H_2_O (18.623 g, 3.13 mol) and 2‐methylimidazole (20.525 g, 12.5 mol) were dissolved in 25 mL of methanol in a 50 mL measuring cylinder to form solution A and B, respectively. To obtain a 50 nm ZIF‐8 sample, the solution A was then poured into B to mix rapidly in a 500 mL bottle, and followed by an addition of 450 mL methanol. This mixture was kept stirring for 1 h. For 250 nm ZIF‐8 sample, the solution B poured into A to mix rapidly and repeat aforementioned process. Both the sample mixture solutions were left undisturbed at room temperature for 24 h. After this, the white precipitate of ZIF‐8 was obtained by methanol washing and centrifugation. The samples were dried at 80 °C.

*Synthesis of ZIF‐8 Samples—ZIF‐8 (Sample 500 nm and 1 µm)*: The large‐sized ZIF‐8 samples were synthesized as follows.^15^, ^24^ Zn(NO_3_)_2_⋅6H_2_O (7.274 g, 2.47 mol) and 2‐methylimidazole (8.111 g, 9.88 mol) were dissolved separately in 50 mL of methanol for each to form solution A and B, respectively. Then these two solutions were rapidly mixed together to form solution C. The ZIF‐8 samples of 500 nm and 1 µm size were obtained by adding a moderator, 1‐methylimidazole of 4.056 g (3.938 mL) and 8.111 g (7.875 mL), respectively, to solution C followed by the addition of 400 mL excess methanol. These solutions were stirred for 1 h and left at room temperature for 24 h. After this, the samples were collected by methanol washing and centrifugation. The samples were dried at 80 °C.

*Synthesis of ZIF‐8 Samples—ZIF‐8 (Sample > 10 µm)*: A solid mixture of zinc nitrate tetrahydrate Zn(NO_3_)_2_⋅4H_2_O (4.2 g, 0.016 mol) and 2‐methylimidazole (1.2 g, 0.015 mol) were dissolved in 360 mL DMF in the 500 mL bottle. The solution was then transferred to two 200 mL Teflon‐lined autoclaves and treated at 140 °C for 24 h in an oven. The mother liquor was removed and the precipitate was washed with DMF followed by chloroform. The precipitate was collected from the upper layer, and dried at high temperature, 200 °C overnight to remove the pore occluded DMF molecules.

*Synthesis of ZDCPs by Pyrolysis*: In all cases, ZIF‐8 (200 mg) was placed in an alumina boat and then transferred into a horizontal tube furnace. The furnace tube was closed with the gas feed‐through end seals, and the sample area was purged thoroughly with nitrogen. The nitrogen flow was maintained throughout the reaction. The carbonization at 900 and 1000 °C was performed separately for 6 h with a heating rate of 5 °C min^−1^. The carbons obtained are named as ZDCP‐900 and ZDCP‐1000. All samples for further characterizations were handled in ambient air and without further any chemical treatment, such as acid treatment or chemical activation.

*Structural Characterization*: X‐ray diffraction (Stoe Stadi‐P, Cu Kα, λ = 1.54056 Å) was carried out by using a 0.5 mm diameter glass capillary filled with the sample. SEM (JEOL 6701) was carried out by pasting the samples on carbon tape. TEM (JEOL 2100) was carried out by drop‐coating the methanol dispersed samples onto a carbon‐coated copper TEM grid. The N_2_ (at 77 K for porosity estimation) and CO_2_ (at 273 K) adsorption–desorption isotherms were obtained by Quantachrome Autosorb‐iQC analyzer. The SSA_BET_ was calculated based on the BET (Brunauer–Emmett–Teller) method. Nonlocal density functional theory method with slit/cylindrical pores was applied to desorption isotherm to obtain pore size distribution, micropore, and cumulative pore volumes. The samples were degassed at 180 °C overnight under dynamic vacuum before the gas adsorption measurements. FTIR data were obtained by Bruker ALPHA FTIR Spectrometer (Platinum‐ATR) with background correction. Raman spectroscopy (using a 514.5 nm laser, Renishaw) was carried out on hand pressed powder samples on a glass slide. XPS (Thermo Scientific, Al Kα) was carried out by samples deposited on the carbon tape. Thermogravimetric analyses (Setaram, Setsys) were carried out up to 1000 °C at a heating rate of 5 °C min^−1^ under argon atmosphere.

*Electrochemical Characterization*: The electrochemical measurements, for supercapacitors and ORR, were carried out on the Autolab (Metrohm PGSTAT302N) electrochemical station at room temperature.

*Supercapacitor Fabrication and Testing*: Working electrodes were prepared by mixing the active carbon material (2.00 mg for dry weight by considering the ultraporous capillary action for moisture adsorption of ≈30 wt%) with additional 10 wt% PTFE (adjusted from diluting the as‐received 60 wt% dispersion in water, Sigma Aldrich) and ethanol until paste‐like, using an agate mortar and pestle, followed by transferring the paste thin‐film onto the current collector (nickel foam disks of 10 mm diameter). The nickel foam disks were cut from a sheet (battery grade from MTI corp.) and then treated with 30% HCl for 5 min in an ultrasonication bath to remove factory/shipping contaminations and surface oxidation, followed by washing and drying. The active carbon‐coated electrodes were dried at 60 °C for a couple of hours and compressed at ≈1 ton using a pelletizer. The symmetric supercapacitor was fabricated by the assembly of two working electrodes and a cellulose membrane separator into a sandwich‐like structure in a stainless‐steel split flat cell (19 mm diameter, MTI corp.) along with the electrolyte (6.0 m KOH) at ambient conditions. In all the cases, the dried electrodes were left soaked in the intended electrolyte at least overnight to 24 h before being subjected to electrochemical tests.

All the supercapacitor tests were carried out by a two‐electrode method on symmetric supercapacitors under aqueous 6.0 m KOH electrolyte at room temperature. Before actual measurements, the supercapacitor was subjected to a number of CV cycles at a scan rate of 50 mV s^−1^ until stable and superimposed CV curves were obtained. Actual CV tests were conducted at different scan rates between (5 and 500) mV s^−1^ in a fixed voltage range of 0.0–0.8 V. The charge–discharge curves with respective upper and lower cut‐off voltages were recorded at a wide range of discrete applied current densities between 0.5 and 25 A g^−1^, on single electrode, 2.00 mg active material base. Specific gravimetric capacitance *C* (F g^−1^) was calculated from GCD curves according to: *C* = 4(*I* × *∆t*)/(*m* × *∆V*), where *I* is the discharge current (A); *m* is the total mass of active material on both the electrodes (g); Δ*t* is the discharge time (s), and Δ*V* is the operating voltage (V); the factor 4 is related to normalization to the mass of one electrode for the two identical capacitors in series. Impedance spectra were recorded in the frequency range of 1 00 000–10 Hz.

*ORR tests* were conducted in a three‐electrode configuration under O_2_ saturated alkaline (0.1 m KOH) electrolyte. The three‐electrode system was consisted of a glassy carbon rotating disk (3 mm diameter) as working electrode, and Pt‐foil (1 cm × 1 cm) and Ag/AgCl/saturated KCl as counter and reference electrode, respectively. The O_2_ saturation was maintained by 30 min pre‐purge and continuous gas bubbling during the measurements. For the preparation of RDE, 2 mg sample was dispersed in a 500 µL suspension which was consisting of 480 µL of deionized water and 20 µL of Nafion solution (5 wt%), then the suspension was sonicated for 30 min or more to form a uniform dispersion of catalyst ink. Then, 5 µL catalyst ink was micropipetted and dropped on the RDE tip followed by drying at 55 °C before the electrochemical measurements. The CV curves were recorded with a voltage scan rate of 100 and 10 mV s^−1^ in the potential range of +0.2 to −0.8 V, and the LSV curves were recorded in the potential range of +0.2 to −0.8 V at a voltage scan rate of 10 mV s^−1^. The ORR LSV curves were measured at a series of rotating speeds (800, 1200, 1600, and 2000 rpm).

The electron transfer number (*n*) was calculated using the Koutecky–Levich equation

(1) $$\frac{1}{J}\;=\;\frac{1}{J_{L}}\;+\;\frac{1}{J_{K}}\;=\;\frac{1}{B\omega^{1/2}}\;+\;\frac{1}{J_{K}}$$

(2) $$B\;=\;0.62nFC_{0}{\left(D_{0}\right)}^{2/3}v^{-1/6}$$

(3) $$J_{K}\;=\;nFkC_{0}$$

where *J* is the measured current density (mA cm^−2^), *J*_L_ and *J*_K_ are the diffusion and kinetic limiting current densities (mA cm^−2^), ω is the angular velocity of the rotary electrode during experiments, *F* is the Faraday constant (96 485 C mol^−1^), *C*_0_ (1.26 × 10^−6^ mol mL^−1^) and *D*_0_ (1.9 × 10^−5^ cm^2^ s^−1^) are the O_2_ bulk concentration and diffusion coefficient in the 0.1 m KOH electrolyte, and ν (0.01 cm^2^ s^−1^) is the viscosity of the electrolyte, *k* is the electron‐transfer rate constant. For the angular velocity *ω*, unit is in rad s^−1^, the unit conversion can follow the relation: rad s^−1^ = (2π × rpm)/60.

The Tafel slopes were determined from the plots of applied potential versus log(current density) of LSV curves measured at 1600 rpm.

## Conflict of Interest

The authors declare no conflict of interest.

## Supporting information

Supplementary
