# Supplementary material for: Size‐Related Electrochemical Performance in Active Carbon Nanostructures: A MOFs‐Derived Carbons Case Study
Source: Adv Sci (Weinh). 2019 Aug 21;6(20):1901517. doi: 10.1002/advs.201901517 (PMC6794624; doi:10.1002/advs.201901517)
Supplement: Supplementary file 1 — Supplementary [file ADVS-6-1901517-s001.pdf]

Copyright WILEY-VCH Verlag GmbH & Co. KGaA, 69469 Weinheim, Germany, 2019.

ADVANCED SCIENCE
Open Access

Supporting Information

for *Adv. Sci.,* DOI: 10.1002/advs.201901517

Size-Related Electrochemical Performance in Active Carbon Nanostructures: A MOFs-Derived Carbons Case Study

Srinivas Gadipelli, Zhuangnan Li, Yue Lu, Juntao Li, Jian Guo, Neal T. Skipper, Paul R. Shearing, and Dan J. L. Brett*

Copyright WILEY-VCH Verlag GmbH & Co. KGaA, 69469 Weinheim, Germany, 2019.

Supporting Information

Size-related electrochemical performance in active carbon nanostructures: a MOFs-derived carbons case study

Srinivas Gadipelli, Zhuangnan Li, Yue Lu, Juntao Li, Jian Guo, Neal T. Skipper, Paul R. Shearing, and Dan J. L. Brett*

Table S1. Literature reported capacitance values of the ZIF-8 derived carbons. The synthesis temperature and capacitance measurement conditions, such as in alkaline or acid electrolyte, two- or three-electrode configurations and corresponding current density or scan rate for the capacitance value are listed.

S/No	SSA_{BET} (m^2 g^{-1})	*C* (F g^{-1})	*T* (°C), time, acid treatment	Size (nm)	Method – 2 or 3 electrode	Electrolyte	at load (A g^{-1}) / [mV s^{-1}]	Ref.
1	1980	245	1000, 8h, -	600	3	1.0M KOH	1	41
2	826	160	1000, 4h, -	250	3	1.0M H_2SO_4	[20]	40
3	472	54	1000, 5h, -	>10000	3	0.5M H_2SO_4	[5]	63
4	822	72	1000, 5h, -	>10000	3	0.5M H_2SO_4	[5]	63
5	1110	200	1000, 5h, -	200	3	0.5M H_2SO_4	[5]	35
6	920	130	950, 5h, HCl	300	2	1.0M H_2SO_4	1	37
7	1132	168	950, - , -	50	3	6.0M KOH	1	36
8	603	150	950, 5h, -		3	KOH	1	38
9	1038	155	950, 5h, -		3	KOH	1	38
10	1470	165	950, 5h, -		3	KOH	1	38
11	983	104	900, 2h, HNO_3	200	2	6.0M KOH	1	31
12	1620	175	900, 2h, -	600	3	6.0M KOH	1	33
13	800	230	915, 4h, HCl	250	3	1.0M H_2SO_4	1	32
14	1118	175	900, 3h, HCl	100	3	6.0M KOH	1	18
15	925	156	900, 3h, HF	500	3	1.0M H_2SO_4	1	64
16	1075	214	900, 5h, HF	200	3	0.5M H_2SO_4	[5]	35
17	679	160	800, 3h, HCl	50	3	1.0M H_2SO_4	1	65

18	825	170	800, 2h, HCl	90	2	6.0M KOH	1	42
19	736	148	800, 2h, HCl	600	2	6.0M KOH	1	42
20	693	128	800, 2h, HCl	1900	2	6.0M KOH	1	42
21	558	107	800, 2h, HCl	200	2	6.0M KOH	1	46
22	847	208	800, 2h, HCl	400	3	6.0M KOH	1	66
23	751	110	800, 3h, HCl	800	3	1.0M H_2SO_4	1	47
24	1499	165	800, 3h, HF	50	3	1.0M H_2SO_4	1	50
25	1111	155	800, 2h, HCl	50	3	6.0M KOH	1	45
26	881	145	800, 2h, HCl	200	3	1.0M KOH	1	67
27	989	201	800, 3h, -	200	3	1.0M H_2SO_4	1	48
28	559	140	800, 1h, HCl	50	3	1.0M KOH	1	68
29	1389	165	800, 5h, HCl	100	3	6.0M KOH	1	60
30	1523	190	800, 5h, HF	>1000	2	1.0M H_2SO_4	1	44
31	1000	140	800, 5h, HCl	500	2	1.0M H_2SO_4	1	43
32	1610	220	800, 4h, HF	1500	3	1.0M H_2SO_4	[5]	49
33	1610	115	800, 4h, HF	1500	2	1.0M H_2SO_4	1	49
34	1057	285	950, 3h, -	300	3	6.0M KOH	1	69
35	773	295	900, 2h, HCl	1000	3	1.0M KOH	1	70
36	820	230	800, - , -	500	3	6.0M KOH	1	71
37	-	145	800, 2h, -	100	3	6.0M KOH	1	72
38	378	115	1000, 3h, -	100	3	6.0M KOH	1	73
39	617	45	800, 4h, HCl	300	2	1.0M H_2SO_4	1	61
40	943	125	800, 5h, HF	2000	2	0.5M H_2SO_4	1	52
41	675	85	950, 1h, -	100	3	KOH	1	74
42	489	175	950, 1h, -	100	3	KOH	1	74
43	898	212	900, 2h, HF	100	3	0.5M NaCl	1	34
44	264	160	800, 3h, HCl	30	3	6.0M KOH	1	75
45	798	161	800, - , -	400	3	1.0M NaCl	[5]	51
46	455	53	750, 5h, -	100	3	6.0M KOH	1	39
47	686	129	850, 5h, -	100	3	6.0M KOH	1	39
48	783	223	950, 5h, -	100	3	6.0M KOH	1	39
49	799	116	1000, 5h, -	100	3	6.0M KOH	1	39
50	406	163	950, 5h, -	100	3	6.0M KOH	1	39
51	566	85	950, 5h, -	100	3	6.0M KOH	1	39
52	515	123	950, 5h, -	100	3	6.0M KOH	1	39
53	640	167	950, 5h, -	100	3	6.0M KOH	1	39
54	1051	63	800, 10h, HCl	100	2	1.0M H_2SO_4	0.25	62
55	1955	107	800, 10h, HCl	100	2	1.0M H_2SO_4	0.25	62

56	1558	125	1000, 3h		3	6.0M KOH	1	55
57	1430	120	1000, 3h		3	6.0M KOH	1	55
MOF Carbons								
58	1861	112	1000, 5h – KOH act@750C, 1h, HCl		3	0.5M H_2SO_4	[5]	63
59	2264	168	1000, 5h – KOH act@750C, 1h, HCl		3	0.5M H_2SO_4	[5]	63
60	1190	235	1000, 2h – KOH act@750C, 1h, HCl	50	3	1.0M H_2SO_4	[5]	76
61								
62	1341	112	800, 10h & KOH activated		2	H_2SO_4	0.25	62
63	2972	251	800, 10h & KOH activated		2	H_2SO_4	0.25	62
64	2491	237	KOH activated		3	6.0M KOH	1	57
65	1386	134	KOH activated		3	6.0M KOH	1	77
66	1880	181	KOH activated		3	6.0M KOH	1	77
67	1244	128	KOH activated		3	6.0M KOH	1	77
68	1764	252	900		2	1.0M H_2SO_4	1	56
69	1576	208	900		2	1.0M H_2SO_4	1	56
70	1713	152	900		2	1.0M H_2SO_4	1	56
71	1408	156					0.5	78
72	682	192	700		3	6.0M KOH	1	53
73	823	175	800		3	6.0M KOH	1	53
74	1115	188	900		3	6.0M KOH	1	53
75	1241	179	1000		3	6.0M KOH	1	53
76	1129	230	700 & act		3	6.0M KOH	1	53
77	1059	225	800 & act		3	6.0M KOH	1	53
78	959	176	900 & act		3	6.0M KOH	1	53
79	909	154	1000 & act		3	6.0M KOH	1	53
80	1147	180			3	6.0M KOH	1	79
81	1084	202	900		3		1	80
82	1290	150						81

83	1269	120						81
84	1812	149	1000					82
85	1543	170						82
86	384	72						82
87	1673	222						82
88	1271	187						82
89	2222	274						82
90	1559	168	1000		2	1.0M H_2SO_4	1	10
91	1492	150	1000		2	1.0M H_2SO_4	1	10
92	1286	100	1000		2	1.0M H_2SO_4	1	10
93	2489	165	1000		3	6.0M KOH	1	83
94	2872	203	KOH act			1.0M H_2SO_4		20
95	2524	149						84
96	3504	161	KOH act					85
97	1328	145				1.0M H_2SO_4	[10]	86
98	1699	143				1.0M H_2SO_4	[10]	86
99	2116	180				1.0M H_2SO_4	[10]	86
100	1397	185				1.0M H_2SO_4	[10]	86
101	880	146				6.0M KOH	1	87
102	1513	159	Act			6.0M KOH	1	87
103	1916	190	Act			6.0M KOH	1	87
104	2926	280	Act			6.0M KOH	1	87
105	2184	170	1000, 3h		2	1.0M H_2SO_4	1	88
106	1378	150	1000, 3h		2	1.0M H_2SO_4	1	88
107	1326	134	1000, 3h		2	1.0M H_2SO_4	1	88
108	920	114	1000, 3h		2	1.0M H_2SO_4	1	88
109	495	110	1000, 3h		2	1.0M H_2SO_4	1	88
110	513	95	1000, 3h		2	1.0M H_2SO_4	1	88
111	1391	119				6.0M KOH	1	89
112	1796	128				6.0M KOH	1	89
113	2137	143				6.0M KOH	1	89
114	2587	146				6.0M KOH	1	89
115	2857	149				6.0M KOH	1	89
116	2587	146					1	90

Additional References

63. Q. Wang, W. Xia, W Guo, L. An, D. Xia, R. Zou, *Chem. Asian J.* **2013**, *8*, 1879.
64. J. Tang, R. R. Salunkhe, H. Zhang, V. Malgras, T. Ahamad, S. M. Alshehri, N. Kobayashi, S. Tominaka, Y. Ide, J. H. Kim, Y. Yamauchi, *Sci. Rep.* **2016**, *6*, 30295.
65. Y. Wang, B. Chen, Y. Zhang, L. Fu, Y. Zhu, L. Zhang, Y. Wu, *Electrochim. Acta* **2016**, *213*, 260.
66. Z. Li, H. Mi, L. Liu, Z. Bai, J. Zhang, Q. Zhang, J. Qiu, *Carbon* **2018**, *136*, 176.
67. D. Y. Chung, K. J. Lee, S.-H. Yu, M. Kim, S. Y. Lee, O.-H. Kim, H.-J. Park, Y.-E. Sung, *Adv. Energy Mater.* **2015**, *5*, 1401309.
68. Y. Wang, B. Chen, Z. Chang, X. Wang, F. Wang, L. Zhang, Y. Zhu, L. Fu, Y. Wu, *J. Mater. Chem. A* **2017**, *5*, 8981.
69. W. Bao, A. K. Mondal, J. Xu, C. Wang, D. Su, G. Wang, *J. Power Sour*. **2016**, *325*, 286.
70. B. Han, G. Cheng, E. Zhang, L. Zhang, X. Wang, *Electrochim. Acta* **2018**, *263*, 391.
71. L. Xin, R. Li, Z. Lu, Q. Liu, R. Chen, J. Li, J. Liu, J. Wang, *J. Electroanal. Chem*. **2018**, *813*, 200.
72. X. Zhang, Q. Fan, H. Yang, H. Xiao, Y. Xiao, *New J. Chem*. **2018**, *42*, 17389.
73. H. Yu, W. Zhu, H. Zhou, J. Liu, Z. Yang, X. Hu, A. Yuan, *RSC Adv*. **2019**, *9*, 9577.
74. Q. Gan, S. Liu, K. Zhao, Y. Wu, Z. He, Z. Zhou, *RSC Adv*. **2016**, *6*, 78947.
75. L. Wan, E. Shamsaei, C. D. Easton, D. Yu, Y. Liang, X. Chen, Z. Abbasi, A. Akbari, X. Zhang, H. Wang, *Carbon* **2017**, *121*, 330.
76. J. Huang, F. Hao, X. Zhang, J. Chen, *J. Electroanal. Chem*. **2018**, *810*, 86.

MOF-carbons

77. H.-H. Duan, C.-H. Bai, J.-Y. Li, Y. Yang, B.-L. Yang, X.-F. Gou, M.-L. Yue, Z.-X. Li, *Inorg. Chem*. **2019**, *58*, 2856.
78. M.-L. Yue, Y.-F. Jiang, L. Zhang, C.-Y. Yu, K.-Y. Zou, Z.-X. Li, *Chem. Eur. J*. **2017**, *23*, 15680.
79. J. Romero, D. Rodriguez-San-Miguel, A. Ribera, R. Mas-Ballest´e, T. F. Otero, I. Manet, F. Licio, G. Abell´an, F. Zamora, E. Coronado, *J. Mater. Chem. A* **2017**, *5*, 4343.
80. M. Shaibani, S. J. D. Smith, P. C. Banerjee, K. Konstas, A. Zafari, D. E. Lobo, M. Nazari, A. F. Hollenkamp, M. R. Hill, M. Majumder, *J. Mater. Chem. A* **2017**, *5*, 2519.
81. Z.-X. Li, X. Zhang, Y.-C. Liu, K.-Y. Zou, M.-L. Yue, *Chem. Eur. J*. **2016**, *22*, 17734.
82. J. Hu, H. Wang, Q. Gao, H. Guo, *Carbon* **2010**, *48*, 3599.
83. P. Wen, Z. Li, P. Gong, J. Sun, J. Wang, S. Yang, *RSC Adv*. **2016**, *6*, 13264.
84. B. Liu, H. Shioyama, H. Jiang, X. Zhang, Q. Xu, *Carbon* **2010**, *48*, 456.
85. H. L. Jiang, B. Liu, Y. Q. Lan, K. Kuratani, T. Akita, H. Shioyama, F. Zong, Q. Xu, *J. Am. Chem. Soc*. **2011**, *133*, 11854.
86. J.-K. Sun, Q. Xu, *Chem. Commun*. **2014**, *50*, 13502.
87. Y. Li, S. Roy, T. Ben, S. Xu, S. Qiu, *Phys. Chem. Chem. Phys*. **2014**, *16*, 12909.
88. H. B. Aiyappa, P. Pachfule, R. Banerjee, S. Kurungot, *Cryst. Growth Des*. **2013**, *13*, 4195.
89. S. Mo, Z. Sun, X. Huang, W. Zou, J. Chen, D. Yuan, *Synth. Met*. **2012**, *162*, 85.
90. D. Yuan, J. Chen, S. Tan, N. Xia, Y. Liu, *Electrochem. Commun*. **2009**, *11*, 1191.

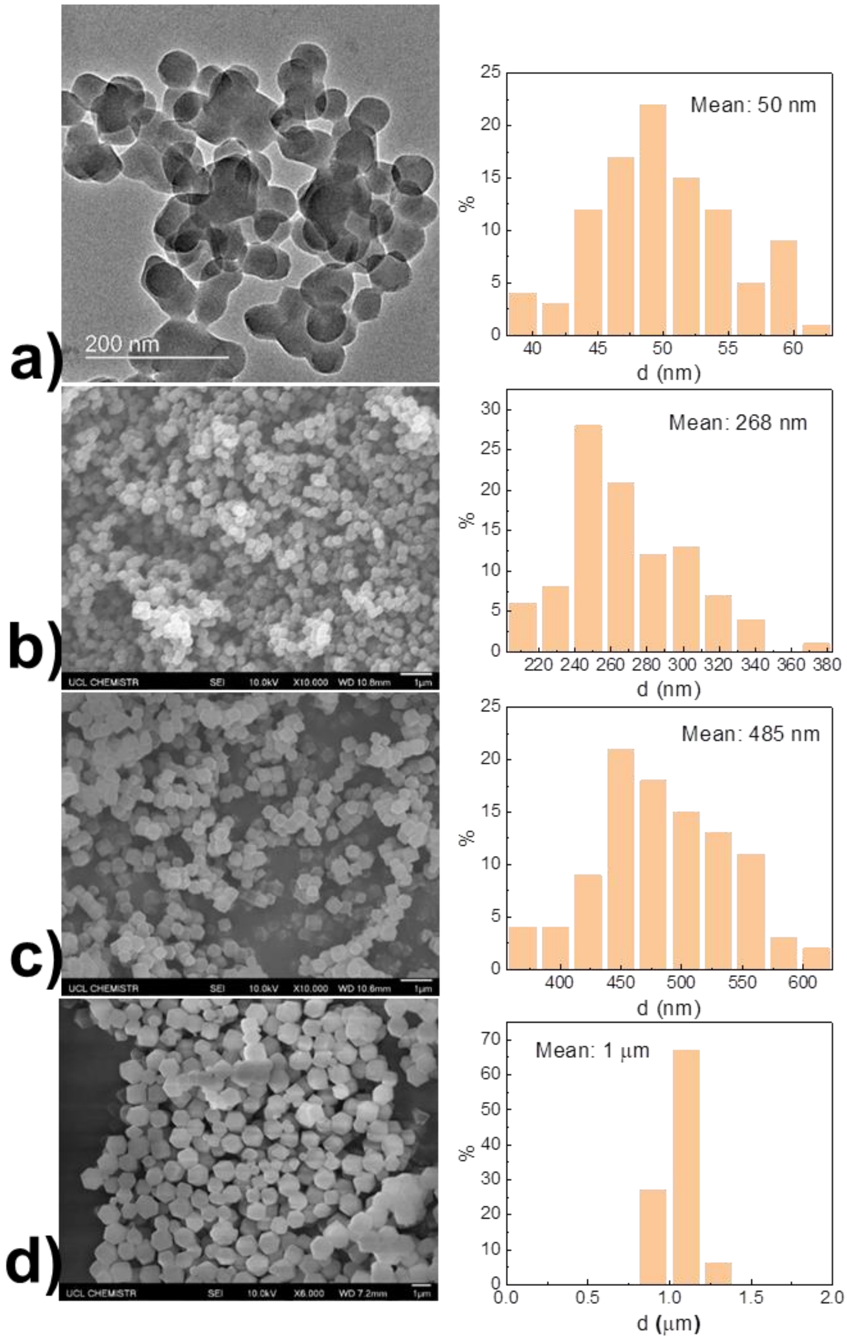

Figure S1. a) TEM, and b-d) SEM images of the ZIF-8 samples of controlled particle sizes. Rows from top to bottom represent the samples of an average 50 nm, 250 nm, 500 nm, 1 micron, respectively. Right panels show corresponding particle size distribution.

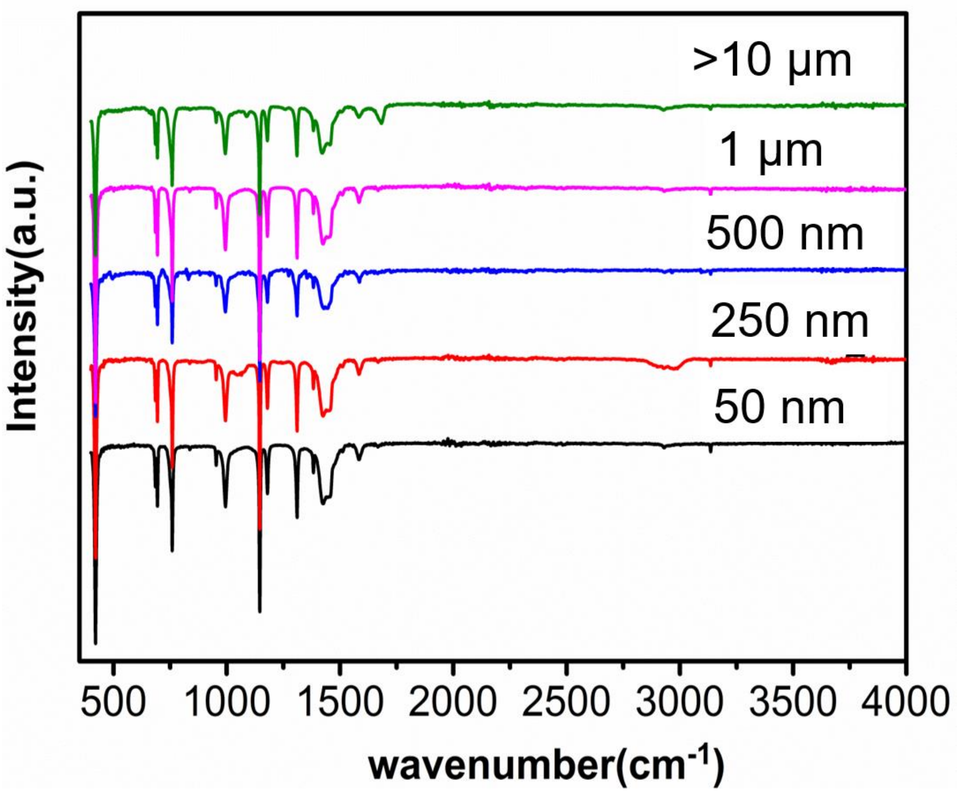

Figure S2. FTIR spectra of ZIF-8 samples of different sizes.

Table S2. N_2 adsorption isotherms derived specific surface area and micropore volume of the ZIF-8 samples.

Sample	SSA_{BET} ($m^2\ g^{-1}$)	Total porosity ($cm^3\ g^{-1}$) at a relative pressure of 0.9 $P\ P_0^{-1}$
ZIF-8-50nm	1800	0.702
ZIF-8-250nm	1820	0.700
ZIF-8-500nm	1840	0.702
ZIF-8-1µm	1880	0.690
ZIF-8->10µm	1440	0.595

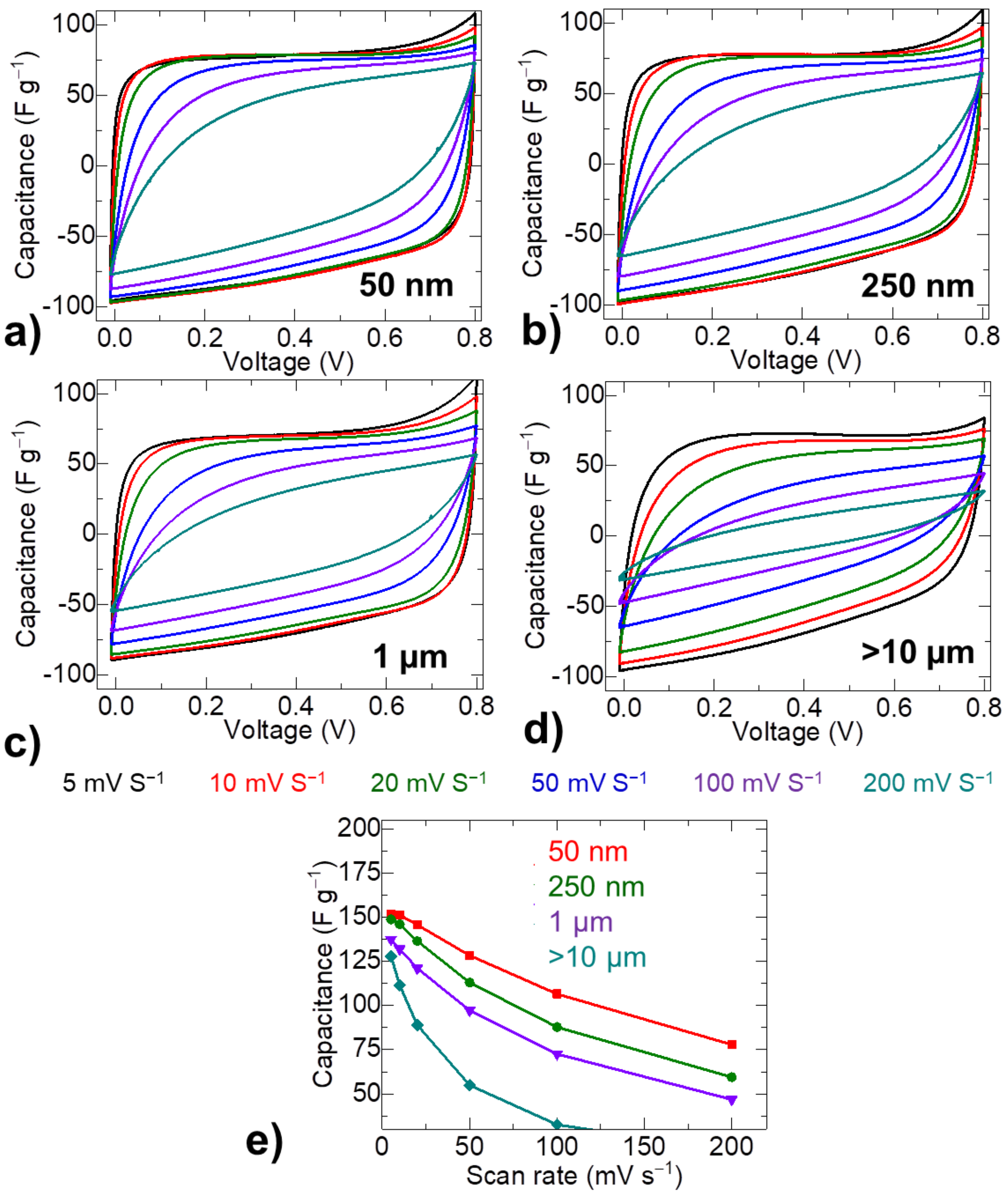

Figure S3. a-d): CV plots of ZDCP-900 series samples of different particle sizes. For each sample CV curves are measured at different scan rates between 5 mV s^{-1} and 200 mV s^{-1}. e): Rate capacitance of the ZDCP-900 samples with respect to the increased scan rate.

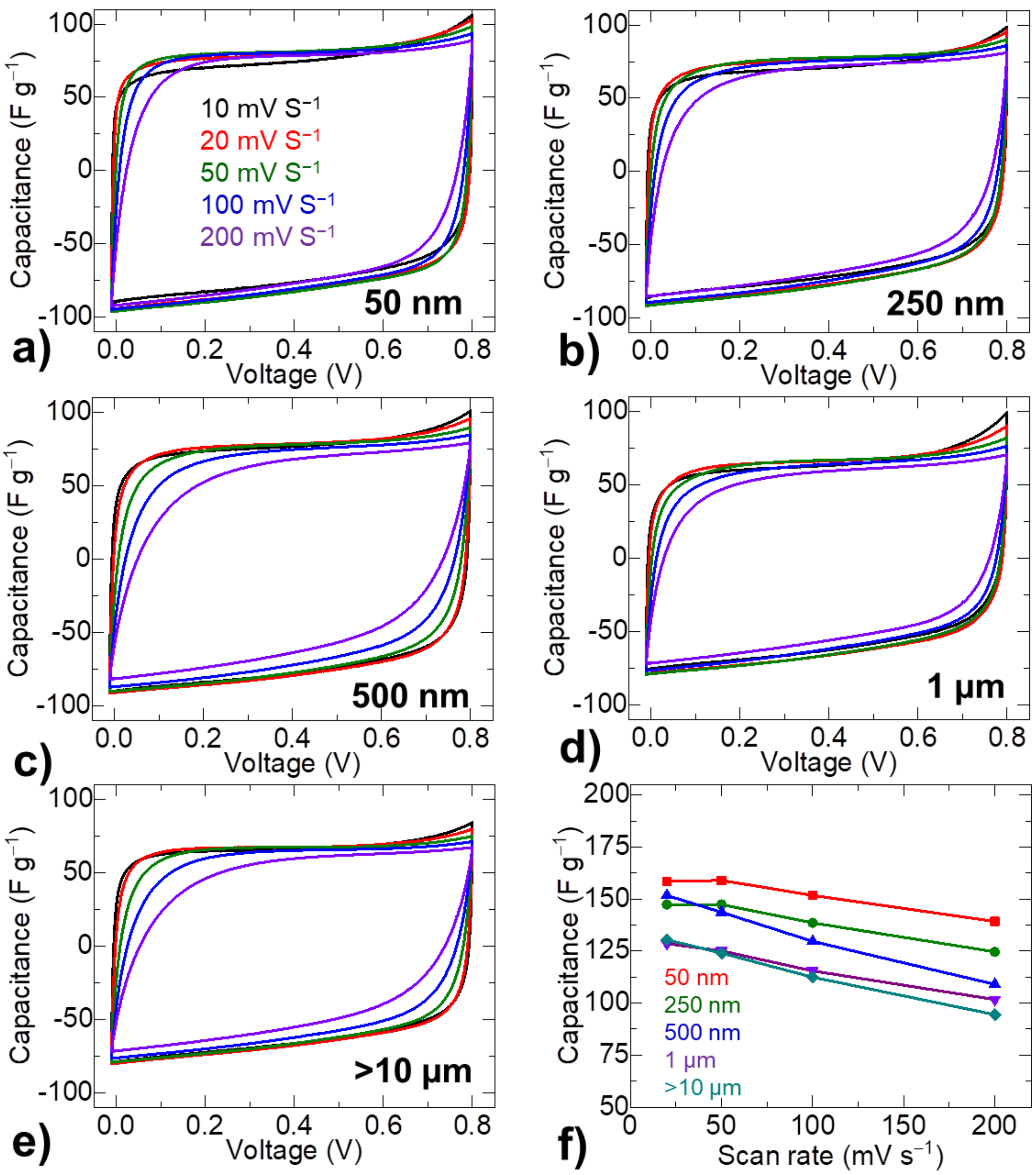

Figure S4. a-e): CV plots of ZDCP-1000 series samples of different particle sizes. For each sample CV curves are measured at different scan rates between 10 mV s^{-1} and 200 mV s^{-1}. f): Rate capacitance of the ZDCP-1000 samples with respect to the increased scan rate.

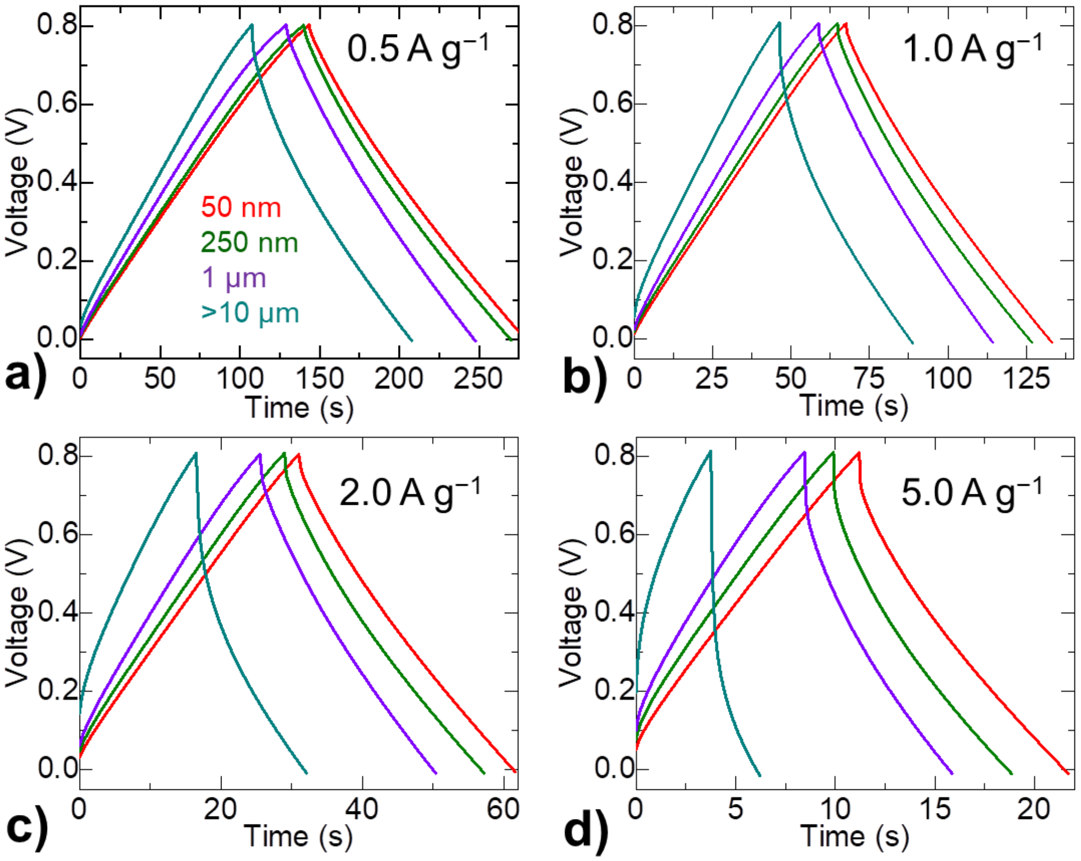

Figure S5. GCD plots of ZDCP-900 series samples of different particle sizes. For each sample GCD curves are measured at different current densities. Samples in the plots are labelled with same colour code.

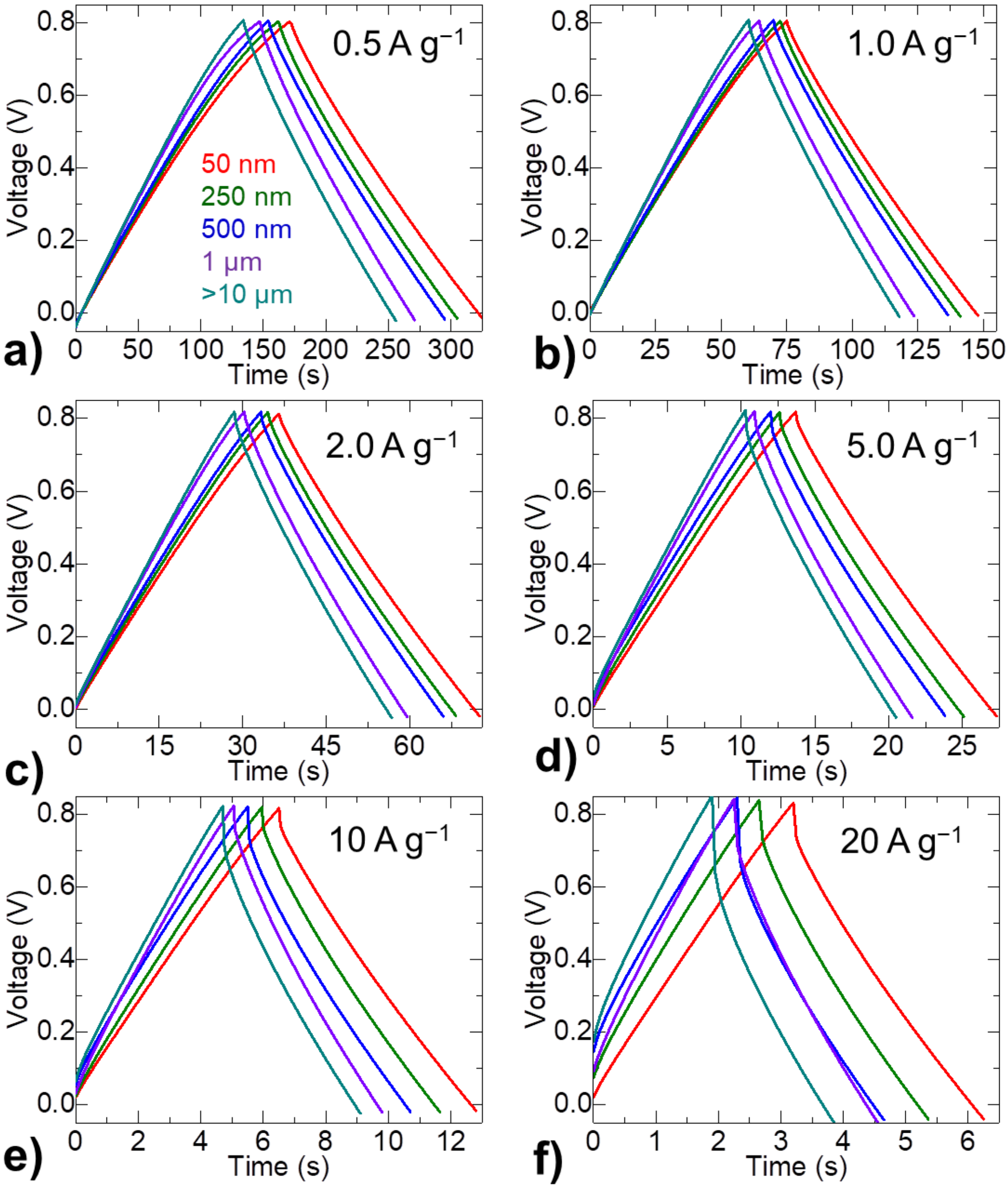

Figure S6. GCD plots of ZDCP-1000 series samples of different particle sizes. For each sample GCD curves are measured at different current densities. Samples in the plots are labelled with same colour code.

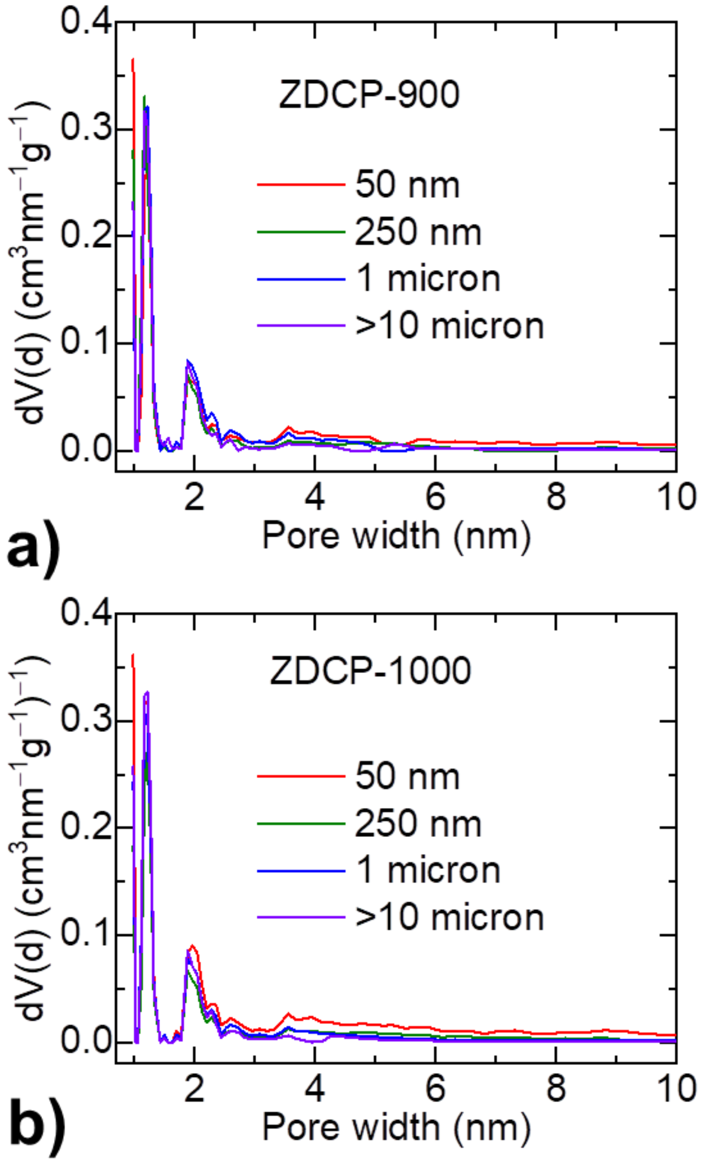

Figure S7. Pore size distribution plots of a) ZDCP-900 and b) ZDCP-1000 series of samples showing a very similar porosity trend.

Table S3. N_2 adsorption isotherms derived specific surface area and porosity: total and micropore volume of the ZDCP samples.

Sample	Specific surface area, SSA_{BET} (m^2 g^{-1})	Total porosity (cm^3 g^{-1}) at a relative pressure of 0.9 P/P_0	Microporosity at ≤1 nm pore-width (cm g^{-1})	Microporosity at ≤2 nm pore-width (cm g^{-1})
Carbonised samples at 900 °C (ZDCP-900)				
ZDCP-50nm	1000	0.470	0.30732	0.36728
ZDCP-250nm	1040	0.456	0.33232	0.39929
ZDCP-500nm	1040	0.458	0.31821	0.39437
ZDCP-1µm	1000	0.423	0.31114	0.38458
ZDCP->10µm	1000	0.480	0.32104	0.36076
Carbonised samples at 1000 °C (ZDCP-1000)				
ZDCP-50nm	1117	0.556	0.32924	0.40631
ZDCP-250nm	1132	0.494	0.3466	0.40545
ZDCP-500nm	1136	0.500	0.35793	0.4295
ZDCP-1µm	1150	0.490	0.37161	0.446
ZDCP->10µm	1070	0.468	0.29428	0.40255

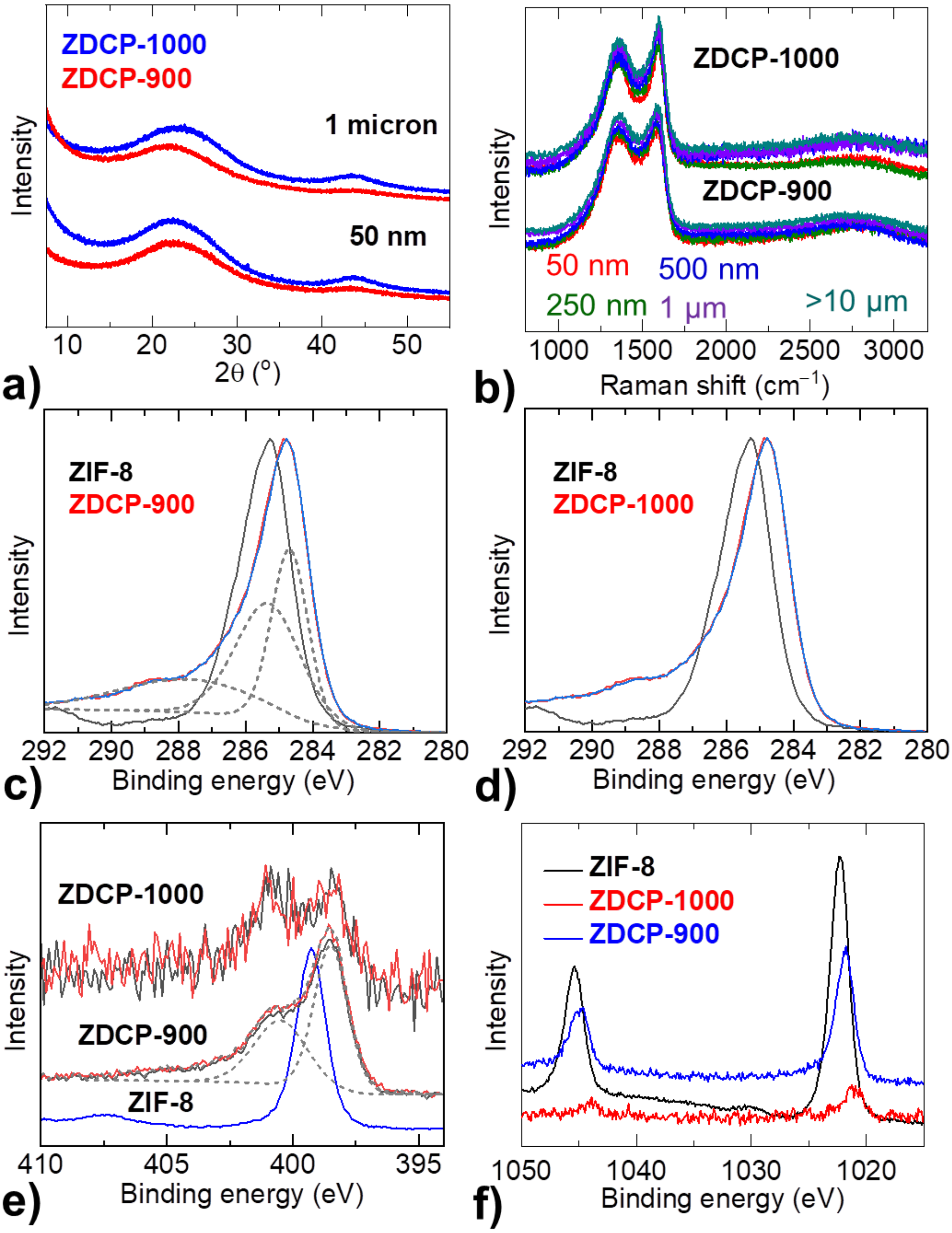

Figure S8. a): PXRD patterns of ZDCPs of different sizes. b): Raman spectra of ZDCPs of different sizes. c-d): XPS C 1s core level spectra of ZDCPs with precursor ZIF-8. The dotted lines show the deconvoluted peaks for ZDCPs. e): XPS N 1s core level spectra of ZDCPs and ZIF-8. The dotted lines show the deconvoluted peaks for ZDCPs. f): XPS Zn 2p core level spectra of ZDCPs and ZIF-8. A shift of the C 1s peak from 285.2 eV, which corresponds to sp^2 C atoms bound to imidazole N atoms, to 284.6 eV for pure graphitic sp^2 C phase in the ZDCPs suggests that there is a considerable loss of ligand N. This is also seen one type symmetric N 1s peak to two-peak in ZDCPs.

Table S4. XPS elemental survey analysis of ZIF-8 and ZDCP-900 and ZDCP-1000 samples.

Sample	Carbon (at%)	Nitrogen (at%)	Zinc (at%)
ZIF-8			
ZIF-8	67.2	27.1	5.7
ZDCP-1000			
ZDCP-50nm	96.50	3.30	trace
ZDCP-250nm	96.35	3.30	trace
ZDCP-500nm	96.60	3.15	trace
ZDCP-1µm	96.00	3.70	trace
ZDCP->10µm	96.80	3.10	trace
ZDCP-900			
ZDCP-250nm	88.80	10.10	1.10
ZDCP-1µm	88.70	10.20	1.10

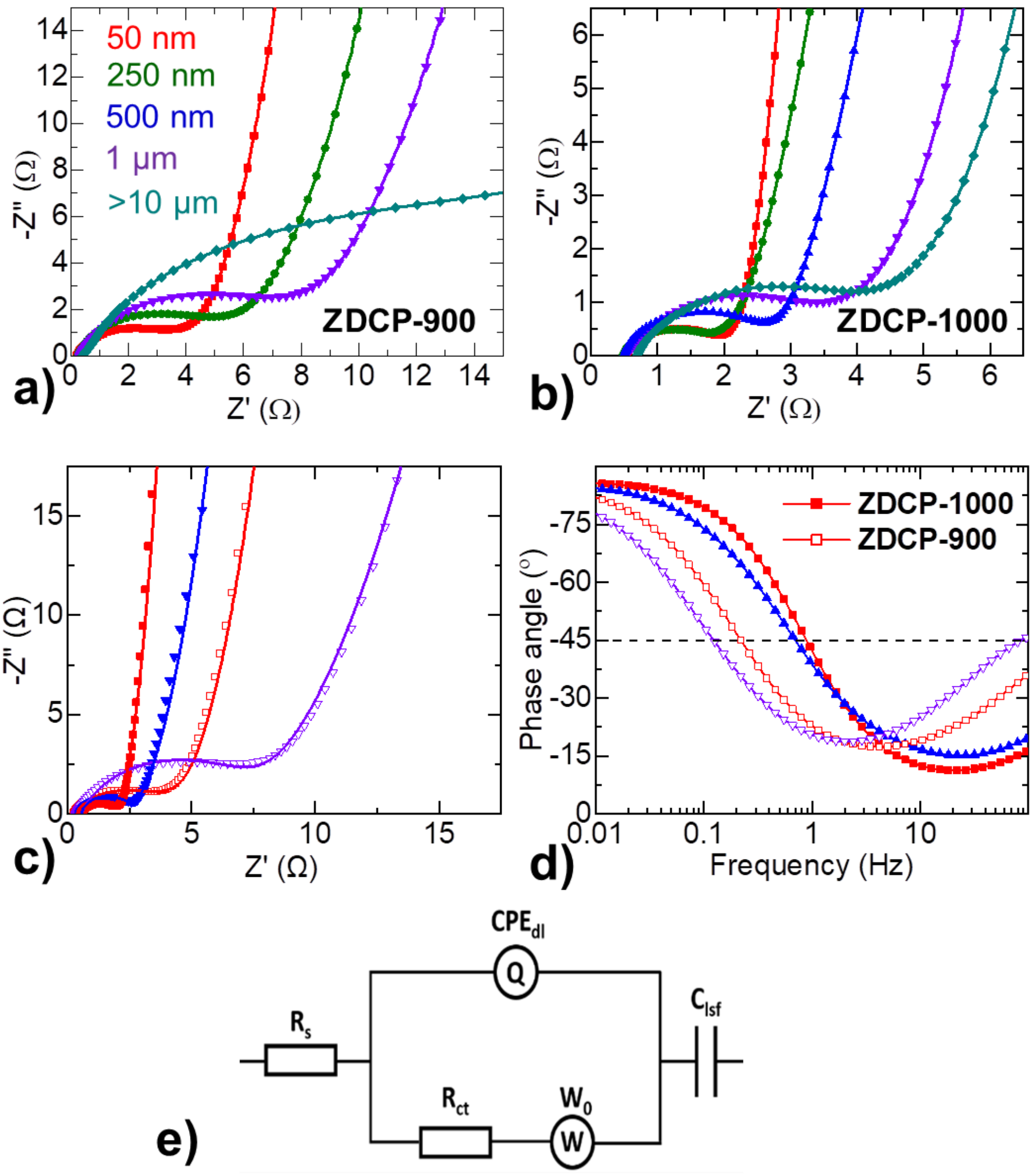

Figure S9. a-b): Impedance Nyquist curves of ZDCPs. c-d): Comparative Nyquist curves of ZDCP-1000 (solid data) and ZDCP-900 (open symbol) samples, corresponding Bode curves, and e): equivalent circuit (the deduced parameter values are summarised **Table S5**). Same colour code applies for the samples in the plots. **R_s:** Combined internal resistance, including the interfacial contact resistance of the material with current collector, the ohmic resistance of electrolyte and the intrinsic resistance of current collector. **R_{ct}:** Interfacial charge transfer resistance, representing the resistance of electrochemical reactions at the electrode surface. **W_0:** Warburg element, describing the transfer and diffusion of the electrons and electrolyte ions in the pores of the electrode materials. **CPE_{dl}:** Constant phase element due to the electrical double-layer behaviour. **C_{lsf}:** Element account for the low-frequency surface capacitance.

Table S5. Equivalent circuit parameters

ZDC electrode	R_s (Ω)	R_{ct} (Ω)	W_0 (mMho $s^{0.5}$)	CPE_{dl} (mMho s^N)	C_{lsf} (mF cm^{-2})
ZDCP-1000 (50 nm)	0.24	1.35	491	0.66 (N=0.91)	102.5
ZDCP-1000 (250 nm)	0.31	1.92	230	1.07 (N=0.87)	86.0
ZDCP-900 (50 nm)	0.49	4.59	183	1.67 (N=0.84)	91.5
ZDCP-900 (1 µm)	0.52	6.82	154	2.28 (N=0.80)	75.4

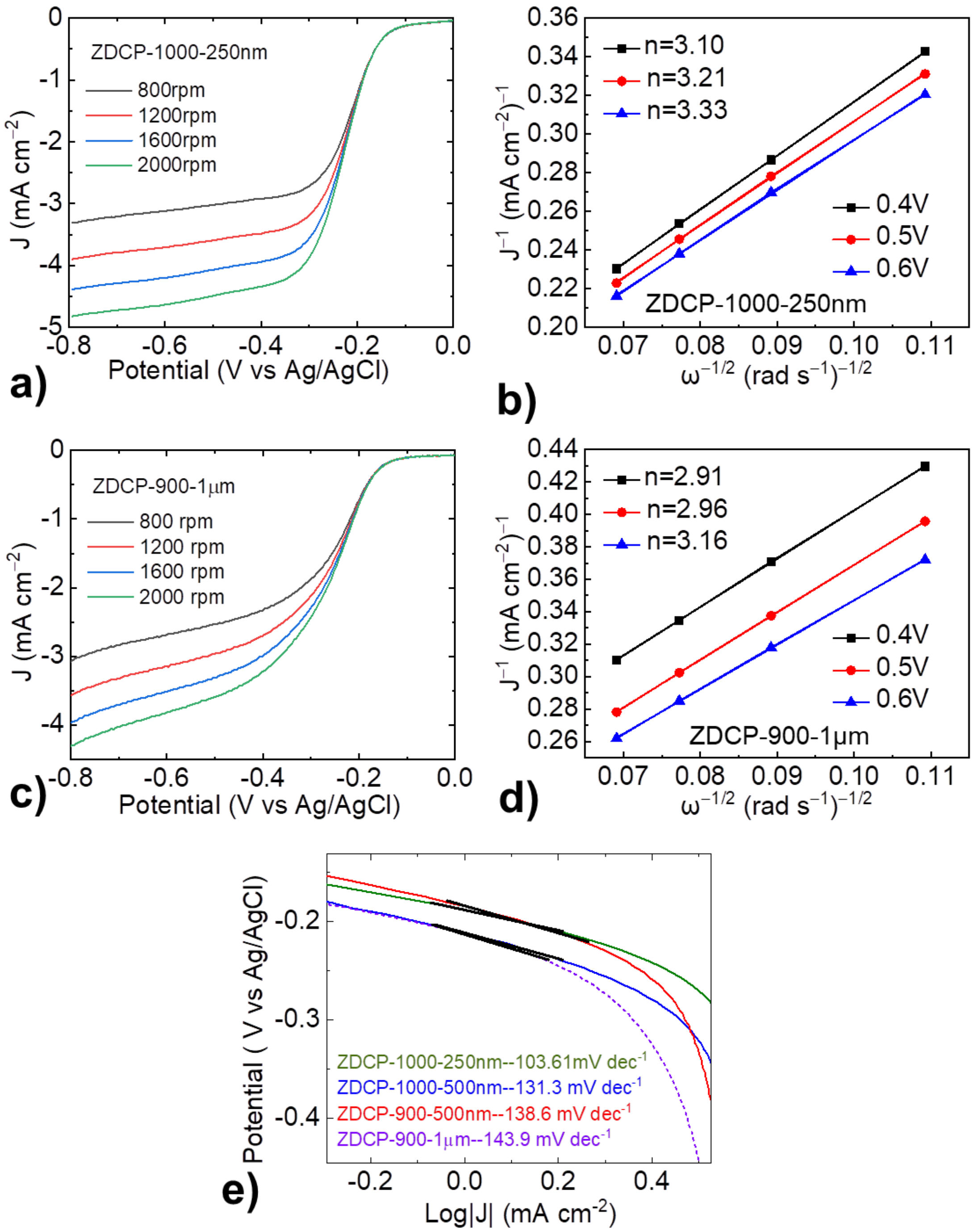

Figure S10. a, c): ORR LSV curves measured at different rotating speeds and b, d): their derived electron transfer number. e): The corresponding Tafel plots and slopes are shown in the bottom plot.
